# Autophagy–Gut Microbiota–Epigenetic Interactions in Disease Pathogenesis: Focus on Neurodegenerative and Neuropsychiatric Disorders and Therapeutic Perspectives

**DOI:** 10.3390/ijms27219502

**Published:** 2026-10-24

**Authors:** Mohammad Amin Mashayekhpour, Hamed Amani, Ali Akbar Hajizadeh Moghaddam, Alireza Moradi, Shabnam Nohesara, Hamid Mostafavi Abdolmaleky

**Affiliations:** 1 Department of Animal Science, Faculty of Science, University of Mazandaran, Babolsar 47416-13534, Iran; mashayekh.amin6775@yahoo.com (M.A.M.); a.hajizadeh@umz.ac.ir (A.A.H.M.); 2 Department of Medical Nanotechnology, Faculty of Advanced Technologies in Medicine, Iran University of Medical Sciences, Tehran 14496-14535, Iran; hamedamani20260@gmail.com; 3 Lung Diseases Research Center, Ardabil University of Medical Sciences, Ardabil 85991-56189, Iran; moradialireza12@gmail.com or ar.moradi@arums.ac.ir; 4 Department of Medicine (Biomedical Genetics), Boston University Chobanian & Avedisian School of Medicine, Boston, MA 02118, USA; 5 Department of Medicine, Division of Gastroenterology, Beth Israel Deaconess Medical Center, Harvard Medical School, Boston, MA 02215, USA

**Keywords:** gut microbiota, neuropsychiatric disorders, autophagy, epigenetics, nutrition

## Abstract

Autophagy is a key cellular process that participates in numerous physiological functions, including maintaining epithelial barrier integrity, regulating intracellular degradation pathways, modulating immune responses, and enhancing antimicrobial defense. Impaired autophagy may contribute to the accumulation of misfolded proteins and insoluble aggregates in the neuronal cytoplasm, defective clearance of damaged organelles and proteins, and the buildup of toxic metabolites. In the gut, these alterations may disrupt the balance between beneficial and potentially harmful microorganisms, contributing to gut dysbiosis, chronic inflammation, and epigenetic alterations in intestinal cells that may indirectly affect the brain. Here, we review the current evidence on the interplay among autophagy, the gut microbiota, and the epigenome in the pathogenesis of diseases, particularly neuropsychiatric disorders (NPDs) and neurodegenerative diseases (NDs). First, we briefly discuss autophagy and its roles in health and disease. We then examine the association between impaired autophagy, inflammation, and epigenetic aberrations in the pathogenesis of NPDs and NDs. Next, we focus on the relationships among gut dysbiosis, defective autophagy, and epigenetic alterations in NPDs and NDs, and discuss microbiota-based therapeutic strategies that may influence epigenetic regulation and autophagic function. Finally, we highlight the current challenges and future directions for investigating the critical role of these interactions in the pathogenesis and treatment of NPDs and NDs.

## 1. Introduction

Neurodegenerative diseases (NDs) and neuropsychiatric disorders (NPDs) are among the leading causes of disability and premature mortality globally [1]. These conditions involve complex interactions among brain circuits, neurotransmitters, inflammation, genetic and epigenetic mechanisms, and environmental factors, and may manifest as overlapping neurological and psychiatric symptoms. Owing to their complex nature and high degree of genetic heterogeneity, these disorders affect millions of people worldwide annually, imposing a significant burden on patients, their families, and society [2]. NPDs include a diverse group of conditions that influence both nervous system function and mental health, giving rise to dysfunction in cognition, emotion, and behavior [3,4]. This broad category encompasses neurodevelopmental disorders, such as autism spectrum disorder (ASD) and major psychiatric disorders, such as schizophrenia (SCZ), bipolar disorder (BD) and major depressive disorder (MDD) [5,6]. NDs mainly include Alzheimer’s disease (AD) and Parkinson’s disease (PD). Despite their distinct pathogenic processes, these disorders often share common clinical symptoms, such as cognitive dysfunction, behavioral abnormalities, and alterations in emotional functioning. NDs are marked by the progressive degeneration and loss of neurons, leading to a gradual decline in cognitive and other neurological functions [7,8]. In contrast, NPDs are primarily linked to disturbances in biological pathways involved in the regulation and production of biomolecules, neurotransmitters, and neuromodulators, which affect mood, cognition, perception, and behavior [9,10,11]. While NDs typically follow a progressive course, with symptom severity increasing over time, NPDs are often chronic or episodic in nature, with substantial variability in symptom intensity and duration.

The gut–brain axis (GBA) is a complex bidirectional communication network linking the gastrointestinal (GI) tract and the central nervous system (CNS) via neural, endocrine, immune, and metabolic pathways [12,13]. This highly integrated system facilitates constant communication between the gut microbiota (GM) and the brain, thereby regulating diverse physiological processes, including brain development, cognition, emotion, behavior, and immune homeostasis [14]. The GM is now recognized as an essential component of the GBA, producing a broad range of bioactive metabolites, neurotransmitters, and neuromodulators that influence neuronal function and brain plasticity [15,16]. Conversely, the brain regulates gut physiology by controlling intestinal motility, secretion, barrier integrity, and immune responses via autonomic and neuroendocrine signaling [17]. Disruption of GBA homeostasis, often characterized by GM dysbiosis, impaired intestinal barrier function, chronic inflammation, and altered microbial metabolite production, has been increasingly implicated in the pathogenesis of NPDs and NDs [18,19,20,21]. These findings have positioned the GBA as a promising therapeutic target for developing interventions aimed at restoring microbial and host homeostasis in brain diseases.

Autophagy is a key process involved in maintaining homeostasis in multiple organs, including the brain and intestine. It contributes to the regulation of interactions between the GM and innate and adaptive immune responses, the degradation of substrates such as aggregated and misfolded proteins and damaged organelles, and host defense against invading pathogens [22,23,24,25]. However, autophagy is considered a double-edged sword with possible therapeutic potential in NPDs and NDs [26,27]. It is not always protective, and its effects may rely on the disease context, cell type, stage, and the extent and period of pathway activation. Although proper autophagic flux plays a key role in maintaining cellular homeostasis and clearing damaged organelles and misfolded proteins, excessive or dysregulated autophagy may aggravate neuronal injury under certain pathological conditions [28]. Impaired autophagy is linked to the pathogenesis of diverse diseases, particularly NPDs and neurodegeneration through diverse mechanisms, including epigenetic mechanisms [29,30,31]. For example, mitochondrial aconitase 2 (ACO2) is a key enzyme in the tricarboxylic acid cycle (Krebs cycle) that connects mitochondrial metabolism with cellular energy homeostasis and autophagy-related processes. Decreased ACO2 activity in PD is linked to the exacerbation of motor deficits and dopaminergic neuron degeneration through the downregulation of autophagy-related genes, such as microtubule-associated protein 1 light chain 3 (LC3) and autophagy-related 5 (ATG5), mediated by reduced histone acetylation at the histone H3 lysine 9 (H3K9) and histone H4 lysine 5 (H4K5) sites [32]. In individuals with ASD, impaired autophagy has been associated with intellectual disability, while experimental evidence has demonstrated that neuronal autophagy-related 7 (ATG7) deficiency causes cognitive deficits in mice [33]. Additionally, chronic 1-methyl-4-phenyl-1,2,3,6-tetrahydropyridine (MPTP) administration has been shown to induce locomotor deficits accompanied by gut dysbiosis, reduced concentrations of the epigenetically active metabolites acetate and propionate, and impaired autophagy in the mouse colon. These alterations were primarily attributed to a reduced abundance of *Bacteroidetes* and an increased abundance of *Firmicutes* [34]. Collectively, these findings suggest a potential interplay among autophagy, GM, and epigenetic regulation in NPDs and NDs. However, direct evidence establishing a three-way mechanistic link remains limited, as many studies have investigated only two components of this relationship.

Therefore, this review critically evaluates the available evidence on the reciprocal interplay among autophagy, GM, and epigenetic regulation across the GBA, with a particular focus on NPDs and NDs. It examines how microbial signals and metabolites may influence autophagy through epigenetic mechanisms and how autophagy dysfunction and epigenetic alterations may, in turn, affect intestinal and microbial homeostasis. Unlike previous studies that have primarily focused on pairwise relationships among these components, this review distinguishes direct evidence of their three-way interactions from pairwise associations and hypothesized mechanisms, highlighting the current limitations in establishing causal relationships. Furthermore, it critically discusses potential therapeutic strategies targeting autophagy and epigenetic regulation, as well as gut microbiota-based interventions, including prebiotics, probiotics, synbiotics, postbiotics, and fecal microbiota transplantation (FMT), while identifying key evidence gaps and future research directions relevant to the pathogenesis and treatment of NPDs and NDs.

## 2. Methods

In this narrative review, we summarized the existing evidence on the interplay among autophagy, the GM, and epigenetic mechanisms, with particular emphasis on their implications for NPDs and NDs. The aim was to integrate findings from different experimental and clinical studies and provide a comprehensive overview of the current knowledge rather than to conduct a formal systematic review or meta-analysis.

A structured literature search was conducted using four electronic databases: PubMed, Web of Science, Scopus, and Embase. Relevant articles published between May 2009 and September 2026 were considered. Representative search terms included “autophagy,” “gut microbiota,” “gut microbiome,” “DNA methylation,” “histone modification,” “histone acetylation,” “microRNA,” “miRNA,” and “epigenetic,” which were combined using appropriate Boolean operators. Additional searches incorporated terms related to GM-based interventions, including probiotics, prebiotics, postbiotics, FMT, and specific diets. Manual screening of relevant publications was performed to identify additional studies that were not retrieved through the electronic database searches.

After initial screening of relevant articles based on their titles and abstracts, full-text assessments of potentially relevant articles were performed. Original clinical, experimental, and observational studies involving human participants, animal models, or in vitro systems and investigating the interactions among autophagy, the GM, and epigenetic regulation were selected for inclusion in the synthesis. Authoritative review articles were also consulted to provide background information, summarize established concepts, and identify relevant seminal primary studies. Publications unrelated to the scope of the review, editorials, and conference abstracts lacking sufficient methodological information were excluded. The evidence was classified according to the level of mechanistic support to facilitate a critical synthesis. This classification included (i) direct evidence indicating interplay among all three components (GM, epigenetics, and autophagy); (ii) pairwise evidence involving two components; and (iii) mechanistic associations inferred by integrating findings from independent studies. Overall, the final narrative synthesis included more than 120 relevant publications.

## 3. An Overview of Autophagy

Autophagic flux encompasses the entire autophagic process, from its initiation to the ultimate lysosomal degradation of cellular components, such as damaged mitochondria and other organelles [22]. Understanding this dynamic sequence provides a basis investigating strategies to modulate neuronal homeostasis and exploring autophagy-targeted interventions for disease treatment [35].

**Figure 1:**
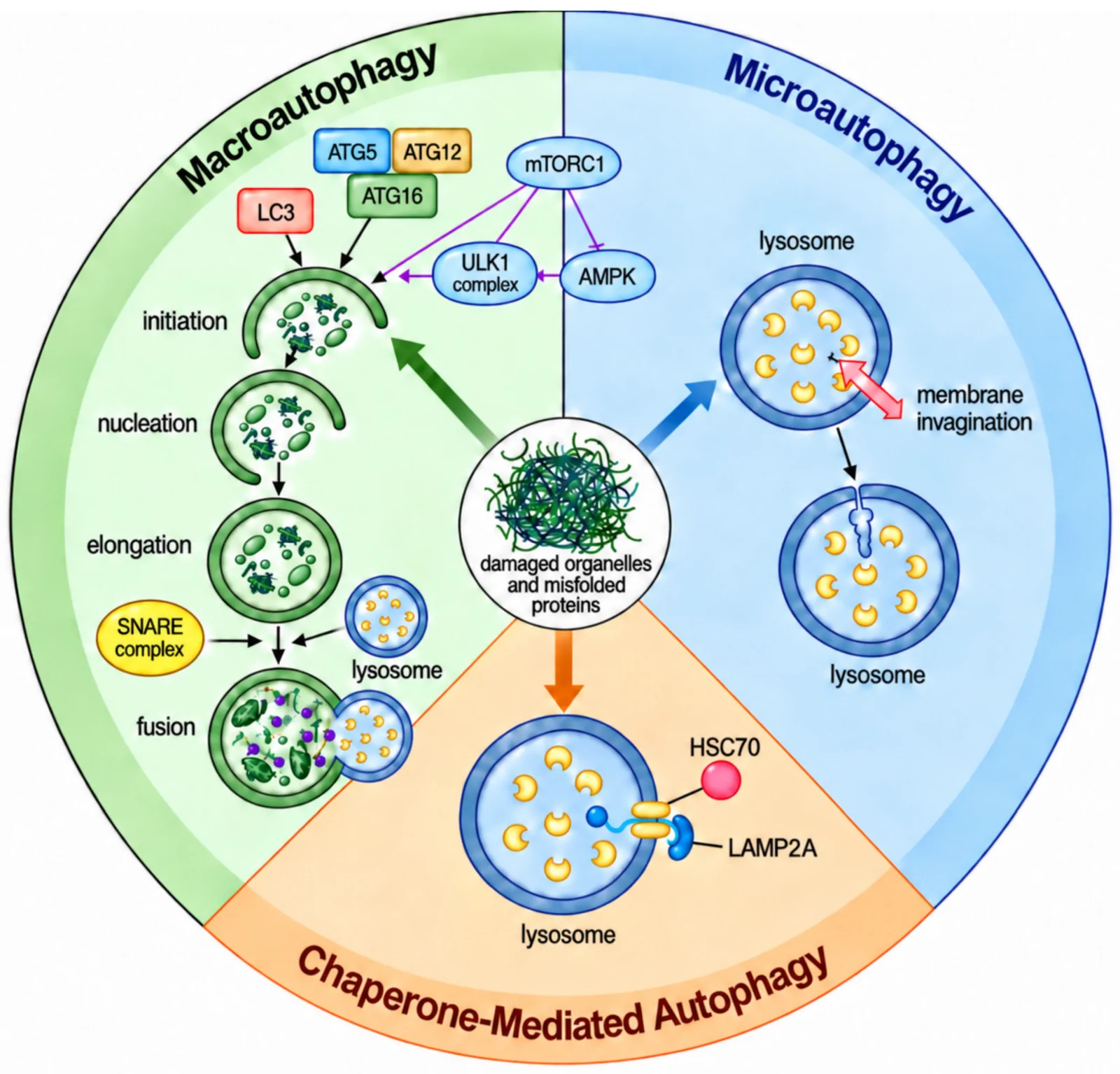
Major representative forms of autophagy, including macroautophagy, microautophagy, and chaperone-mediated autophagy (CMA). They contribute to maintaining cellular homeostasis by facilitating the delivery of cytoplasmic components to lysosomes for degradation. Macroautophagy involves initiation and phagophore formation, cargo sequestration and autophagosome maturation, followed by autophagosome–lysosome fusion and lysosomal degradation. Microautophagy directly engulfs cytoplasmic material via lysosomal membrane invagination or protrusion. CMA selectively recognizes soluble cytosolic proteins containing KFERQ-like motifs via heat shock cognate 71 kDa protein (HSC70) and delivers them to lysosome-associated membrane protein 2A (LAMP2A) for lysosomal translocation and degradation. The figure was created using Microsoft PowerPoint 2019 in combination with Adobe Photoshop 2024 (Version 25.12.0.806).

There are three major classes of autophagy, including macroautophagy, microautophagy, and chaperone-mediated autophagy (CMA) (Figure 1), along with several specialized forms.

### 3.1. Macroautophagy

Macroautophagy is the most commonly investigated type of autophagy and is known as the main cellular recycling system [36]. The process is triggered by cellular stressors, such as nutrient deprivation, oxidative stress (OS), or organelle impairment, which activate AMP-activated protein kinase (AMPK) and inhibit mechanistic target of rapamycin (mTOR) signaling [24]. These changes promote activation of the unc-51-like autophagy-activating kinase 1 (ULK1) complex and the subsequent recruitment of the Beclin 1 (BECN1)-containing class III phosphatidylinositol 3-kinase complex, thereby promoting phagophore formation [37]. The phagophore elongates and then engulfs damaged organelles and biomolecules like misfolded proteins, impaired mitochondria, and other damaged cellular components. Adaptor proteins, such as sequestosome 1 (p62/SQSTM1), mediate selective cargo sequestration by linking ubiquitinated cargo to microtubule-associated protein 1 light chain 3-II (LC3-II) molecules associated with the expanding membrane [38]. Upon membrane closure, a double-membrane vesicle named the autophagosome is generated, which traffics to and fuses with lysosomes to form an autolysosome. Within the autolysosome, lysosomal hydrolases contribute to the degradation of the enclosed organelles and biomolecules, and the resulting breakdown products are recycled to support cellular metabolism and homeostasis [39]. Autophagy plays a pivotal role in development, immunity, aging, and cellular quality control, and its dysregulation has been implicated in NDs. As autophagic flux includes the whole progression of autophagy, from cargo isolation to lysosomal degradation and recycling [40], the efficiency of autophagic function depends not only on the quantity of autophagosomes but also on the effective completion of the entire degradative pathway. Importantly, an elevation in autophagosomes or autophagy-related proteins alone does not necessarily indicate enhanced autophagic flux, as these alterations may also reflect impaired lysosomal degradation. Therefore, both autophagosome formation and subsequent lysosomal degradation should be considered when assessing functional autophagic flux.

In NDs, autophagic flux is frequently disrupted during autophagosome maturation or lysosomal degradation, leading to the accumulation of autophagic vesicles and toxic protein aggregates, increased cellular stress, and potentially contributing to neuronal damage and disease progression [41,42]. However, the specific stage and extent of impairment may vary across diseases, cell types, and disease stages.

Mitophagy is a selective type of macroautophagy that contributes to maintaining cellular homeostasis by targeting dysfunctional and damaged mitochondria for autophagic degradation. Because dysfunctional mitochondria can be important sources of reactive oxygen species (ROS), their selective removal through mitophagy helps maintain mitochondrial quality and can reduce mitochondrial ROS production under conditions of cellular stress [43,44,45]. Therefore, mitophagy is associated with reduced mitochondrial oxygen consumption and ROS production, while supporting the maintenance of cellular and metabolic homeostasis [46]. There is a bidirectional relationship between mitophagy and OS [47]. On the one hand, OS may contribute to the initiation of mitophagy. On the other hand, effective mitophagy may reduce ROS generation and OS by eliminating damaged or dysfunctional mitochondria. In NDs, such as PD, postmortem studies and genetic or toxin-induced models of the disease have demonstrated dysregulated mitophagy, which is associated with elevated ROS generation and OS. Mechanistically, parkin RBR E3 ubiquitin protein ligase (PRKN; also known as parkin)-mediated mitophagy contributes to the selective removal of damaged mitochondria from cells, and mitochondrial impairment is considered a core hallmark of PD pathogenesis [48].

### 3.2. Microautophagy

Microautophagy is an autophagic process in which the lysosome directly engulfs small portions of cytoplasm through the invagination, protrusion, or septation of its own membrane [49]. There is no need for autophagosome formation in this process. Microautophagy is responsible for the continuous turnover of cellular components and contributes to the preservation of cellular quality control and metabolic balance [50]. Although microautophagy occurs under normal conditions and contributes to cellular maintenance and adaptation, it may also participate in the response to stress.

### 3.3. Chaperone-Mediated Autophagy (CMA)

CMA is a selective type of autophagy that is capable of targeting and degrading individual soluble proteins [51]. Proteins targeted for CMA possess a specific amino acid sequence motif recognized by the cytosolic chaperone protein heat shock cognate 71 kDa protein (HSC70), which is capable of binding to these proteins and transporting them to the lysosomal membrane [52]. At the lysosomal membrane, targeted proteins are subjected to a sequence of events that leads to their degradation, including interaction with the receptor lysosome-associated membrane protein 2A (LAMP-2A), unfolding, and direct translocation across the lysosomal membrane into the lysosomal lumen [53]. CMA plays a key role in protein quality control, cellular stress responses, and metabolic regulation, and impaired CMA is linked to aging and various diseases, including neurodegenerative diseases [54,55].

## 4. Interaction Between Epigenetic Mechanisms and Autophagy in the Pathogenesis of NPDs and NDs

Autophagy and its dysregulation are influenced by multiple epigenetic mechanisms, including DNA methylation, histone modifications, and microRNA (miRNA)-mediated post-transcriptional regulation [56,57]. These mechanisms can modulate the expression and activity of key autophagy-related genes and, consequently, influence autophagic flux and cellular homeostasis [58]. In this section, we briefly describe the major epigenetic mechanisms involved in the regulation and impairment of autophagy, providing a foundation for understanding their roles in disease pathogenesis and their interactions with GM-derived metabolic, nutritional, and inflammatory factors. The findings in this section predominantly establish links between epigenetic mechanisms and autophagy; they should not be taken as direct evidence of a three-way interaction with the GM.

### 4.1. DNA Methylation

DNA methylation is one of the key epigenetic mechanisms capable of regulating gene expression through modifications of chromatin architecture and accessibility [59]. This process involves the covalent addition of a methyl group (–CH_3_) to the 5-carbon position of cytosine residues, predominantly at cytosine–phosphate–guanine (CpG) dinucleotide sites and, to a lesser extent, at cytosine–phosphate–adenine (CpA) sites (particularly in the brain), without altering the underlying DNA sequence [60]. Epigenetic modifications, especially DNA methylation, are closely linked to disease development by influencing the regulation of autophagy-related genes and other cellular processes [61]. It has been shown that exposure to stress and environmental perturbations may trigger AD-like pathological changes by altering DNA methylation and inducing aberrant ryanodine receptor-mediated calcium (Ca^2+^) signaling in the mouse brain, with these changes being associated with lysosomal dysfunction, impaired autophagic degradation, and disrupted autophagic flux in neurons [62]. Aging is also linked to hypermethylation of the promoter regions of ATG5 and LC3B in mouse macrophages, which is associated with reduced gene expression and may impair autophagy-related processes and contribute to neurodegenerative conditions [63]. Given that these age-related findings were observed in mouse macrophages, their relevance to autophagy dysfunction in the human brain requires further investigation.

In PD, emerging evidence suggests that the gastrointestinal tract may serve as an early site of α-synuclein pathology. Disturbances in the autophagy–lysosome pathway (ALP), a key cellular system involved in protein degradation and clearance, are associated with the accumulation and aggregation of α-synuclein, which may be linked to epigenetic aberrations. In this context, Gordevicius et al. reported distinct DNA methylation changes across ALP-related genes [64]. In their study, deep sequencing analyses demonstrated aberrant methylation at hundreds of cytosine sites influencing several ALP genes, with a predominance of hypermethylation patterns observed in the brains of PD patients. They also found that chronic intestinal inflammation in mice could cause DNA methylation alterations in ALP genes, and these changes were markedly amplified by α-synuclein pathology [64]. These combined human findings of altered DNA methylation in PD, together with preclinical animal evidence, link intestinal inflammation and α-synuclein pathology to changes in DNA methylation. However, they do not, by themselves, establish that these methylation changes directly cause impaired autophagic flux. Notably, amyloid-beta (Aβ)-induced toxicity in AD also impairs autophagy-related pathways, with these effects being mediated by downregulation of BECN1, ATG5, ATG7, ESR1 (estrogen receptor 1), and ESR2 (estrogen receptor 2), and corresponding DNA hypermethylation of ESR1, ESR2, and ATG7 genes, which can be restored by PaPE-1 (a synthetic nonsteroidal estrogen receptor ligand) treatment [65]. As will be discussed in the following sections, a number of GM-derived metabolites and probiotics may exert similar effects. Other studies reporting the interplay between DNA methylation and autophagy, which may contribute to the pathogenesis of NPDs and NDs, are summarized in Table 1.

### 4.2. Histone Modifications and Modification of Non-Histone Proteins

As fundamental epigenetic regulators, histone modifications play key roles in modulating a broad spectrum of pathophysiological processes in humans [66]. Chromatin-associated post-translational modifications, including histone phosphorylation, ubiquitination, methylation, and acetylation, can regulate gene transcription and thereby influence autophagy-related pathways [67,68]. For example, histone acetylation can modulate autophagic function and autophagosome formation by regulating the expression of key components of the ULK1, BECN1-phosphatidylinositol 3-kinase catalytic subunit type 3 (PIK3C3), and LC3 complexes [69]. In rats, increased expression of the Notch1 receptor and acetylation of H3K9 have been linked to impaired autophagic function and depressive-like and anxiety-like behaviors induced by maternal separation [70].

PD-linked environmental factors have also been found to increase histone deacetylase (HDAC) degradation and histone acetylation in dopaminergic neurons through autophagy, representing an epigenetic dysregulation mechanism implicated in PD pathogenesis [71]. Therefore, the use of HDAC-modulating compounds may potentially help restore HDAC-regulated gene expression and mitigate cellular stress responses and autophagic dysfunction [72]. As an example, Baek et al. found that a newly discovered class II HDAC inhibitor could improve depressive-like behaviors in mice by inducing autophagic activity in microglia, suppressing nitric oxide production, and inhibiting microglial activation and inflammation in the brain [73]. These findings provide preclinical evidence for a potential interaction between histone regulation and autophagy; however, the reported associations do not necessarily establish that histone acetylation directly causes the behavioral phenotypes, and the therapeutic relevance of these effects in human NPDs or NDs remains to be established.

In addition to histone modifications, several studies have demonstrated that acetylation or deacetylation of non-histone proteins can regulate autophagy. Unlike histone modifications, these mechanisms do not directly constitute epigenetic modifications but instead represent post-translational regulation of pre-existing proteins. This mechanism should therefore be distinguished from chromatin-based epigenetic regulation, even though it may interact with epigenetic pathways through shared regulatory enzymes and substrates. For example, BECN1 acetylation at specific lysine residues has been found to disrupt autophagic flux and may contribute to the pathogenesis of AD [74]. Similarly, tubulin deacetylation mediated by sirtuin 2 (SIRT2) or histone deacetylase 6 (HDAC6) activation in the cytosol can reduce microtubule stability and promote microtubule depolymerization, thereby facilitating Tau dissociation and Tau phosphorylation and influencing autophagic flux in AD animal models [75]. These findings highlight the importance of distinguishing non-histone protein acetylation/deacetylation from chromatin-based epigenetic regulation. Because HDAC6 and SIRT2 can act on non-histone substrates, their effects on autophagy should not automatically be interpreted as evidence of altered histone modifications or gene transcription.

Overall, these findings indicate that chromatin-based epigenetic mechanisms, particularly histone modifications, can regulate autophagy-related gene expression, whereas acetylation/deacetylation of non-histone proteins provides an additional layer of post-translational regulation of autophagy. More examples of the interactions between DNA methylation, histone modifications, and autophagy-related genes involved in the pathogenesis of PD, AD, and NPDs are summarized in Table 1. These studies vary in scope, with most animal and cell-based studies investigating the underlying mechanisms of epigenetic dysregulation of autophagy-related genes, whereas human studies primarily report associations between epigenetic alterations and NPDs or NDs.

**Table 1:** DNA methylation, histone modification changes, or non-histone post-translational modifications of autophagy-related genes in neurodegenerative and neuropsychiatric diseases.

Disease/Epigenetic Alteration	Type of Study/Samples	Affected Autophagy Genes/Main Methods	Key Findings	Ref.
Parkinson’s disease (PD)/DNA methylation	Mouse/primary cortical and mesencephalic neurons/cells and brain tissues	Ambra1, Parkin, and LC3B/Whole-genome bisulfite sequencing and western blotting (WB)	Hypermethylation-low expression of Ambra1 gene exacerbate DA neuron injury both in vitro and in vivo through Ambra1/Parkin/LC3B-mediated mitophagy pathway	[76]
PD/DNA methylation	Rats and SH-SY5Y cells/dopaminergic neurons in substantia nigra	PINK1 and DJ-1 (PARK7) genes/Bisulfate sequencing and qRT-PCR	CpG hypermethylation induced downregulation of PINK1 and DJ-1 genes and exacerbated dopaminergic neuronal loss following long-term exposure to airborne particles	[77]
PD/histone modification	Idiopathic PD and G2019S-LRRK2 cells/fibroblasts from PD subjects with or without G2019S mutation and controls	Histone acetyltransferase (HAT), histone deacetylase (HDAC) and mitophagy genes/WB and RT-PCR	Idiopathic PD showed reduced clearance of defective mitochondria and mitophagy followed by more ROS production due to imbalanced total HATs and HDACs activities	[78]
PD/histone modification	Rotenone-induced model in rats/Striatum	BECN1, p62, LC3, Bcl-2 ratio, cytochrome c, and caspase-3/RT-PCR and WB	Striatal HDAC6 reduced α-synuclein clearance via inhibition of the ubiquitin proteasome system and autophagy	[79]
PD/histone modification	Rotenone-treated SH-SY5Y cells	α-syn, LC3, and p62/immunofluorescence and WB	Upregulation of HDAC4 in PD substantia nigra and locus coeruleus increased α-syn accumulation by inhibiting autophagy	[80]
Alzheimer’s disease (AD)/DNA methylation	Mouse microglia cells	MEF2A and other autophagy-related genes/Microarray, single-nucleus RNA-seq and qRT-qPCR	Hypermethylation of MEF2A enhancer decreases the expression of MEF2A and autophagy-related genes	[81]
AD/non-histone post-translational modification	APP/PS1 mice/brain	Transcription factor EB (TFEB), p62, ATG2A, and ATG5/RT-PCR, WB, Elisa	Acetylation of TFEB, as a mechanism of TFEB activation enhanced the expression of lysosomal and autophagic genes and mitigated AD pathogenesis	[82]
AD/histone modification	Aβ1–42-induced SH-SY5Y cells	FSTL1, LC3, Bcl-2, P62, cleaved cas- pase-3, BECN1, IκBα, p-IκBα, p65, P300, RUNX1 and NF-κB/WB, Chromatin immunoprecipitation and RT-PCR	FSTL1 overexpression by H3K27 acetylation through P300/RUNX1 co-activation intensifies autophagy-mediated neuronal injury and NF-κB-dependent inflammation	[83]
Autism spectrum disorder (ASD)/DNA methylation	Five ASD patients vs. seven controls/Postmortem brain tissues	RABGGTB/RT-PCR and whole genome methylation profiling	Promoter hypomethylation of CpG islands of RABGGTB, a gene associated with autophagy and synaptic function increases its expression	[84]
ASD/non-histone post-translational modification	Mouse/hippocampus	FoxO1 by SIRT2/WB and immunofluorescence	Autophagic activity caused by increasing the acetylation of FoxO1 using SIRT2 gene deletion reduces neuronal richness and increases neuroinflammation	[85]
Major depressive disorder (MDD)/histone modification	Lipopolysaccharide (LPS)-induced depression-like mouse model/hippocampus	PINK1, Parkin, and PTEN/WB, RT-PCR and immunofluorescence	Astrocyte-specific upregulation of HDAC7 disturbed PTEN-induced putative kinase 1 (PINK1)-Parkinson protein 2 (Parkin)-related mitophagy, leading to decreased mitochondrial ATP release, neuronal injury and depression	[86]
Psychosis/DNA methylation	Patients with recent-onset psychosis and controls/peripheral blood	ETFA, TYRO3P, RNF31, PSME2, NUMBL, ATG4B, MARCH1, ANP32C, IGF2BP2, and METTL16/whole genome methylation profiling	20 differentially methylated sites in patients vs. controls; association between hypomethylation of cg15028047 and reduced expression of autophagy-related genes	[87]
Schizophrenia/DNA methylation	Human/patient-derived and control-derived cell lines	PSMD5, AEN, FAM20B and LRRN4/whole genome methylation profiling	Hypomethylation of AEN involved in controlling genotoxic stress-induced autophagy	[88]

### 4.3. miRNAs

miRNAs, a class of endogenous non-coding RNAs typically about 21 nucleotides in length, act as key post-transcriptional regulators of gene expression during health and disease [89,90]. Numerous miRNAs may contribute to the pathogenesis of NPDs and NDs due to their pivotal roles in regulating mitochondrial function and the autophagy pathway [91]. The severity or development of diseases may be mediated by interactions among the GM, autophagy, and cellular miRNA profiles [92]. However, direct evidence linking all three components within the same experimental system, particularly in NPDs and NDs, remains limited, and many studies are based on pairwise or indirect evidence. For example, *Fusobacterium nucleatum*–derived extracellular vesicles have been found to mediate colitis severity in mice via miR-574-5p/CARD3–dependent autophagy activation [93]. Adherent-invasive *E. coli* (adherent-invasive *Escherichia coli* (AIEC)), a harmful bacterium, has also been found to increase the expression of miRNAs (miR-30c and miR-130A) targeting genes involved in the autophagy response (ATG5 and ATG16L) in mouse enterocytes, which in turn aggravates intestinal inflammation [94]. These findings demonstrate interactions among the GM, miRNAs, and autophagy in intestinal disease models, but do not, by themselves, establish a corresponding mechanism in NPDs or NDs.

Nevertheless, autophagy can also regulate the miRNA-induced silencing complex (miRISC) by controlling the expression of the key component AGO2 [95]. Genetic and chemical impairment of autophagy, or exposure to microbial products, such as *Helicobacter pylori* vacuolating cytotoxin A (VacA) toxin, may elevate Argonaute RISC component 2 (AGO2) expression in epithelial and immune cells, including intestinal epithelial cells as well, leading to an altered miRNA profile [95]. These dysregulated miRNAs are enriched in pathways involved in different diseases, highlighting the possibility that GM-mediated modulation of autophagy may alter miRNA-mediated gene regulation and contribute to intestinal inflammation and potentially influence disease onset or progression.

In AD, miR-299-5p has been shown to regulate neuron survival programs by modulating autophagy. Specifically, miR-299-5p reduces ATG5 expression and alters autophagy-related activity in primary neurons of mice with the “amyloid precursor protein Swedish mutation/presenilin 1 deletion of exon 9” (APPswe/PS1dE9), as well as in Neuro-2a (N2a) and SH-SY5Y cells [96]. Additionally, miR-140 overexpression has been reported in AD model rats and neurons, whereas its inhibition reduces mitochondrial dysfunction and is associated with changes in autophagy-related markers, including decreased mTOR expression and phosphorylation and increased BECN1 and LC3-II/LC3-I levels, along with reductions in β-amyloid, p-Tau (Ser396), p-Tau (Thr231), and Tau. These effects were associated with the upregulation of PTEN-induced kinase 1 (PINK1), a direct target negatively regulated by miR-140 [97]. It is also worth noting that changes in LC3-II/LC3-I and BECN1 do not, by themselves, establish increased autophagic flux; these findings should therefore be interpreted as evidence of altered autophagy-related processes rather than definitive evidence of enhanced autophagic activity. As another example, mitochondrial miR-204 has been found to be a key player in ROS-mediated autophagy and neuronal damage in triple-transgenic Alzheimer’s disease (3×Tg-AD) model mice, and its silencing has been reported to improve disease-related outcomes [98]. Duan et al. also found that miR-124 aggravates depressive-like symptoms in chronic unpredictable mild stress (CUMS) mice by targeting Ezh2 in the hippocampus and is associated with changes in autophagy-related markers, including increased LC3-II/I, p62, and ATG7 expression [99]. It should also be noted that the simultaneous increase in LC3-II/I and p62 may reflect altered autophagosome accumulation or impaired autophagic degradation, rather than increased autophagic flux.

In psychotic disorders, a systematic review of 10 studies found increased expression of miR-181b-5p and miR-34a-5p as the most consistent findings in SCZ spectrum disorders across the brain and blood cells [100]. It has been shown that miR-181b modulates autophagy-related processes in a rat epilepsy model, with changes in LC3-II/I, BECN1, ATG5, ATG7, and ATG12, through the toll-like receptor 4 (TLR4)–p38 mitogen-activated protein kinase (p38 MAPK)/c-Jun N-terminal kinase (JNK) pathway [101] while miR-34a can also inhibit autophagy-related activity by decreasing high mobility group box 1 (HMGB1) expression [102]. However, in ASD, downregulation of miR-181a-5p has been reported in blood cells [103]. Notably, lipopolysaccharide (LPS)-induced systemic inflammation decreases the expression of miR-181a/b/c/d in microglia, whereas restoration of miR-181 family members attenuates microglial activation [104]. Because these findings derive from different disorders, tissues, and experimental settings, they should not be interpreted as evidence of a uniform role for the miR-181 family across NPDs.

Table 2 summarizes other studies investigating the interplay between miRNAs and autophagy in the pathogenesis of NPDs and NDs. Several miRNAs listed in Table 2 that regulate autophagy-related genes in these disorders have been reported to be influenced by the GM or GM-derived metabolites. Examples include miR-9, miR-21, miR-124, and miR-125b [105]. LPS, which may increase during gut dysbiosis, also upregulates the expression of miR-124, miR132, miR-134, and miR-155 in the rat brain [106]. Although these findings do not directly demonstrate that GM-induced changes in these miRNAs mediate autophagy-related effects in NPDs or NDs, they indirectly support potential mechanisms linking GM or GM-derived metabolites, miRNAs that regulate autophagy-related genes, and autophagy (the GM–miRNA–autophagy axis) together.

## 5. Interaction Between Autophagy and the GM

The GM and autophagy exhibit a bidirectional relationship, in which the GM regulates autophagic activity, while alterations in autophagy influence GM-associated mechanisms involved in the pathogenesis of various diseases [122,123,124]. A preclinical study demonstrated that GM dysbiosis, following chronic sleep deprivation, could induce NLR family pyrin domain containing 3 (NLRP3)-mediated autophagic dysfunction, glycogen synthase kinase 3 beta (GSK-3β) activation, and tau hyperphosphorylation in the hippocampus, changes associated with cognitive decline in mice [125]. Although this study demonstrates an association between GM dysbiosis, altered autophagy and neurodegenerative-related pathology, it does not by itself establish a direct epigenetic mechanism.

**Figure 2:**
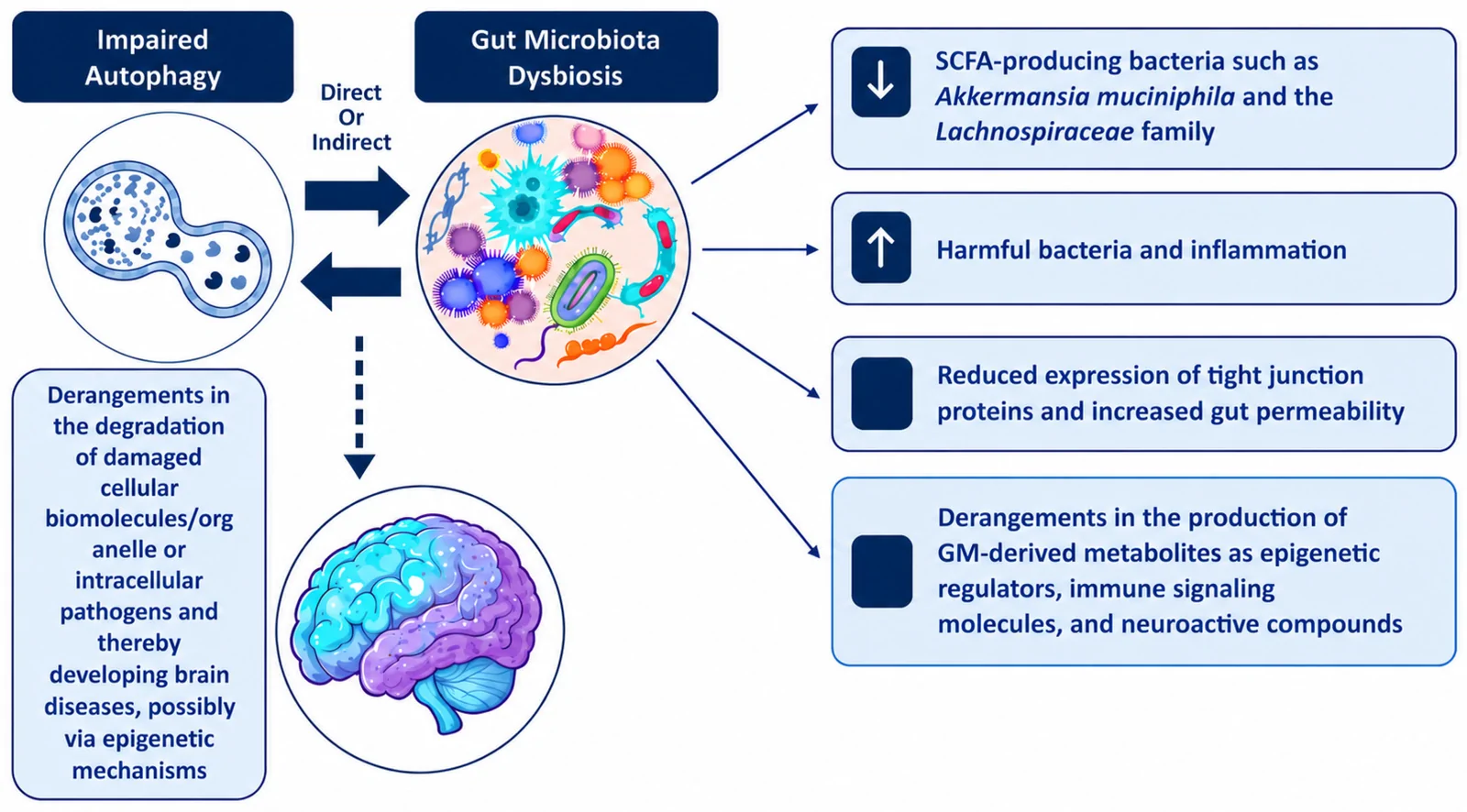
The bidirectional interplay between impaired autophagy and gut microbiota (GM) dysbiosis in the pathogenesis of neuropsychiatric disorders (NPDs) and neurodegenerative diseases (NDs). Impaired autophagy may alter the gut microbiota composition and host immune responses by reducing the abundance of short-chain fatty acid (SCFA)-producing bacteria, while promoting the expansion of potentially pathogenic or inflammation-associated microorganisms. Gut microbial dysbiosis can also impair autophagic activity through inflammatory and other signaling pathways, with potential involvement of epigenetic mechanisms. This interaction may impair the clearance of damaged cellular biomolecules, organelles, or intracellular pathogens, thereby contributing to pathophysiological processes relevant to NPDs and other brain diseases involving epigenetic mechanisms. Solid arrows indicate relationships directly supported by the cited evidence, whereas dashed arrows indicate proposed, indirect, or mechanistically inferred relationships. The downward arrow (↓) indicates a decrease, while the upward arrow (↑) indicates an increase. The figure was created using Microsoft PowerPoint 2019 in combination with Adobe Photoshop 2024 (Version 25.12.0.806).

Gut dysbiosis also plays a key role in autophagy dysregulation and short-chain fatty acid (SCFA) transport and absorption. In a cellular model, co-culture of Caco-2 cells with *P. gingivalis* caused disruption of cellular junctions by impairing autophagic function, particularly via the ATG5-LC3 pathway, resulting in reduced expression of tight junction proteins and diminished SCFA absorption, with reported effects involving monocarboxylate transporter 1 (MCT1) and sodium-coupled monocarboxylate transporter 1 (SMCT1), along with the G-protein-coupled receptor 43 (GPR43)/G-protein-coupled receptor 109A (GPR109A) pathway [126]. As an in vitro intestinal model, this study demonstrates associations between microbial exposure, autophagy-related pathways, and SCFAs but should not be directly extrapolated to neuronal disease. Conversely, impaired autophagy has been associated with changes in GM composition and host immune responses, including reduced abundance of potentially beneficial bacteria such as *Akkermansia muciniphila* and members of the SCFA-producing *Lachnospiraceae* family, alongside possible increases in potentially pathogenic bacteria [127]. This complex and dynamic interaction affects the performance of the GBA, mediated by SCFAs and other players that can modulate epigenetic machinery [128,129]. Figure 2 illustrates how the bidirectional interplay between impaired autophagy and GM dysbiosis may contribute to the pathogenesis of NPDs and NDs through reduced SCFA production and increased inflammation, both of which can influence epigenetic mechanisms.

The GM affects autophagy via several interconnected molecular pathways, mainly mediated by GM-derived metabolites, immune signaling molecules, and neuroactive compounds, as demonstrated in models of herpes simplex encephalitis and in vitro studies using a Caco-2 monolayer intestinal epithelial barrier model [130,131]. Commensal gut bacteria generate SCFAs through the fermentation of dietary fibers, which act as HDAC inhibitors, and may regulate cellular energy homeostasis through AMPK/mTOR signaling, a major regulatory pathway of autophagy. However, the effects of SCFAs on autophagy are not necessarily uniform and may vary depending on the cellular and physiological context. For example, although SCFAs have been reported to activate AMPK and inhibit mTOR signaling, thereby potentially promoting autophagy, the direction and magnitude of these effects may depend on cell type, SCFA concentration, exposure duration, cellular metabolic state, receptor signaling, and pathological conditions. Propionate-induced autophagy has been associated with increased AMPK activity and diminished mTOR activity in human colon cancer cells [132]. However, these findings should not be directly extrapolated to normal intestinal tissues or the CNS. Conversely, another study reported that SCFAs exerted protective effects on Caco-2 intestinal barrier function in association with autophagy inhibition [130]. These apparently divergent findings suggest that SCFAs may either promote or suppress autophagy depending on the experimental context. Therefore, although SCFAs may contribute to the regulation of autophagy and cellular homeostasis, their effects should be interpreted in a context-dependent manner, and evidence for direct SCFA-mediated regulation of autophagy in neural tissues remains limited. Conversely, pathogen-associated molecular components, including bacterial lipopolysaccharides and peptidoglycan fragments, have the capacity to activate host innate immune receptors, such as Toll-like receptors (TLRs) and nucleotide-binding oligomerization domain (NOD)-like receptors. Activation of these receptors modulates autophagy-dependent processes, including the recruitment of autophagy-related proteins, LC3-II lipidation, and autophagosome formation, which contribute to the selective degradation of damaged cellular biomolecules, organelles, and intracellular pathogens [133]. In addition, the metabolism of dietary tryptophan by the GM produces various bioactive catabolites, such as indole derivatives, which are capable of passing through the intestinal barrier and regulating neuronal signaling pathways relevant to autophagy, inflammation, and OS responses [134]. Interactions between these metabolites and molecular targets like the aryl hydrocarbon receptor (AhR), as well as other signaling pathways, may contribute to maintaining neuronal health and CNS homeostasis [135]. Collectively, the existing evidence indicates that autophagy may serve as a potential mechanistic interface linking the GM with host immune, metabolic, and neurological functions, and that these interactions are bidirectional. Nevertheless, many studies remain pairwise or mechanistically inferred, and direct evidence of a complete GM–autophagy–epigenetic pathway in NPDs and NDs remains limited.

## 6. Gut Microbiota–Targeted Interventions for Modulating Autophagy via Epigenetic Mechanisms

**Figure 3:**
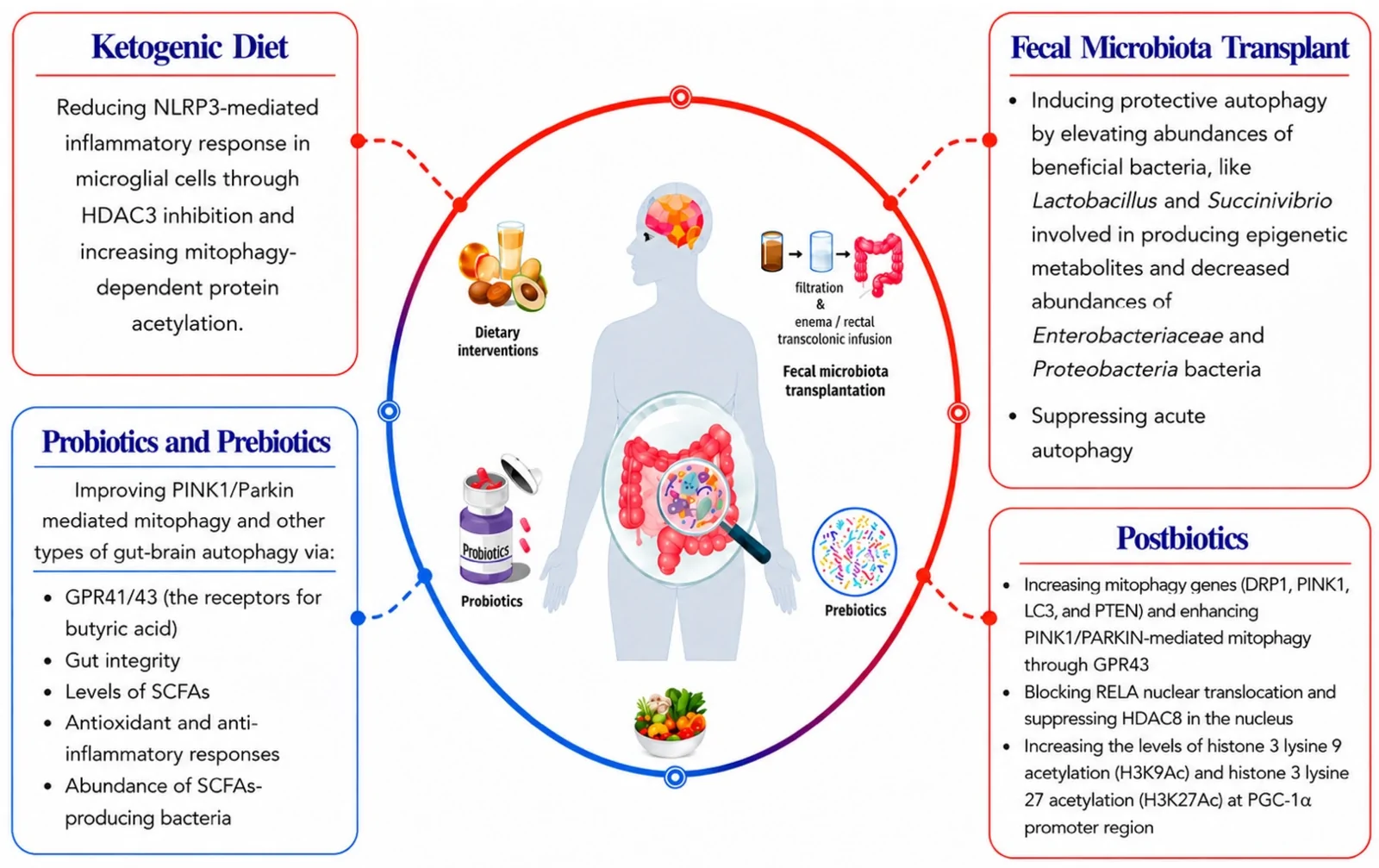
Proposed mechanisms by which microbiota-based interventions, including prebiotics, probiotics, postbiotics, and fecal microbiota transplantation, may alleviate gut inflammation and potentially improve disease-related outcomes in neuropsychiatric disorders (NPDs) and neurodegenerative diseases (NDs) by modulating epigenetic processes and autophagic pathways. by simultaneously modulating epigenetic processes and autophagic pathways. These interventions may promote gut microbial homeostasis, increase the production of beneficial microbial metabolites, reduce intestinal and systemic inflammation, and improve gut barrier integrity, thereby influencing gut–brain axis signaling and potentially contributing to neuroprotection. The depicted interactions represent proposed mechanisms supported by direct, pairwise, or mechanistically inferred evidence, and should not be interpreted as evidence of an established causal pathway linking the GM, autophagy, and epigenetic mechanisms in NPDs or NDs. Abbreviations: Short-chain fatty acids (SCFAs); Histone deacetylase 3 (HDAC3); NLR family pyrin domain containing 3 (NLRP3); Free fatty acid receptor 3 (FFAR3, also termed GPR41); Microtubule-associated protein 1A/1B-light chain 3 (LC3); Peroxisome proliferator-activated receptor gamma coactivator 1-alpha (PGC-1α); Phosphatase and tensin homolog deleted on chromosome ten (PTEN); PTEN-induced kinase 1 (PINK1). The figure was created using Microsoft PowerPoint 2019 in combination with Adobe Photoshop 2024 (Version 25.12.0.806).

The complex crosstalk among the GM, autophagy, and epigenetic regulation in NPDs highlights these interconnected mechanisms as potential therapeutic targets. Strategies involving prebiotics, probiotics, and postbiotics may reshape the GM, attenuate inflammation, regulate autophagic processes, and modulate disease-associated epigenetic changes, thereby potentially contributing to disease prevention and treatment [136]. However, it should be noted that much of the available evidence is derived from preclinical or in vitro models, and only a limited number of studies have demonstrated that these interventions simultaneously affect all three components. Figure 3 depicts various dietary and GM-based interventions that may influence autophagy and epigenetic alterations, highlighting their potential therapeutic relevance for the prevention and management of NPDs and NDs. However, at present, the clinical efficacy of these approaches and the extent to which their effects are mediated by coordinated changes in the GM, autophagy, and epigenetic regulation remain insufficiently established.

### 6.1. Probiotics

Probiotics are live microorganisms capable of exerting health benefits when consumed in adequate amounts by the host [137]. Friendly or commensal microbes present in fermented foods have traditionally been used as supplements for gut health, with proper adherence and safety [138,139].

Emerging evidence has demonstrated that probiotics may contribute to improving neurological function and intestinal injury by modulating autophagy, potentially improving autophagic flux, and diminishing OS-induced disorders and apoptosis through p38 mitogen-activated protein kinase (p38 MAPK)-mediated autophagy (including changes in LC3 and BECN1 and p62 degradation) [140,141]. Reported changes in autophagy-related markers, including LC3, BECN1, and p62, provide evidence of altered autophagy-related processes, but do not necessarily establish p38 MAPK-mediated changes in autophagic flux. The evidence linking probiotic administration to enhanced autophagy also remains heterogeneous, partly because different studies have assessed distinct components of the autophagic pathway and used different experimental models. In particular, increases in LC3 or autophagy-related proteins do not necessarily indicate increased autophagic flux, as accumulation of these markers may also result from impaired lysosomal degradation. Therefore, findings based primarily on LC3, BECN1, p62, or autophagy-related gene expression should be interpreted cautiously unless supported by direct measurements of autophagic flux. It should be noted that the evidence discussed in this section is predominantly preclinical, derived from cell-based experiments and animal models, and should not be interpreted as establishing clinical efficacy in humans.

For example, Haririzadeh Jouriani et al. demonstrated that a probiotic containing several *Lactobacillus* species, namely, *L. plantarum* PR42 and PR365, *L. rhamnosus* PR195, *L. brevis* PR205, and *L. reuteri* PR100, exhibited anti-inflammatory and autophagy-modulating properties in an in vitro inflammatory model using HT-29 cells [142]. Although these findings support a potential relationship between probiotic exposure and autophagy in intestinal epithelial cells, the in vitro nature of the model limits extrapolation to the intact intestine or to neural tissues. Moreover, the effects of a defined multi-strain preparation may not be directly attributable to individual bacterial species or strains. Importantly, this study provides evidence for a probiotic–autophagy interaction but does not establish a direct three-way interaction involving epigenetic regulation.

Hawrysh et al. monitored 49 probiotic strains and examined different stages of mitophagy to clarify whether probiotics have the ability to enhance mitophagy in cells undergoing mitochondrial stress. Their cell-based assays showed that two probiotics, *Saccharomyces boulardii*, a butyrate-producing yeast, and *Lactococcus lactis*, were capable of enhancing mitochondrial PRKN recruitment, phospho-ubiquitination, and MFN degradation. According to their findings in a Drosophila model of PD, administration of these two probiotics led to improvements in longevity and motor function by enhancing lysosomal degradation of impaired mitochondria in the brain [48]. The neuroprotective effects were associated with promoting PINK1/Parkin mediated mitophagy and attenuating OS. This study therefore combines in vitro mechanistic evidence with in vivo evidence from a Drosophila model. The cell-based experiments support the proposed mitochondrial mechanisms, whereas the fly experiments provide organism-level evidence for effects on longevity and motor function. These findings provide stronger mechanistic evidence for effects on mitochondrial quality control because multiple steps of the PINK1/Parkin pathway and lysosomal degradation were examined. However, this study primarily demonstrates a probiotic–mitophagy relationship rather than a direct three-way interaction involving epigenetic regulation.

Nevertheless, the use of a Drosophila PD model and cell-based mitochondrial stress models represents an important source of biological heterogeneity compared with mammalian models of PD. Differences in host physiology, GM composition, and probiotic metabolism may therefore influence the magnitude and mechanism of the observed effects. In fact, neither the cell-based experiments nor the Drosophila model is sufficient to establish therapeutic efficacy in humans with PD.

Wang et al. used engineered *Clostridium butyricum*-pMTL007-GLP-1 to treat a mouse model of PD and found that the treatment alleviated motor dysfunction and neuropathological alterations, elevated TH (tyrosine hydroxylase) expression, and diminished the expression of α-syn by reducing the relative abundance of *Bifidobacterium* at the genus level, enhancing gut integrity, upregulating GPR41/43 (receptors for SCFAs), increasing PINK1/Parkin-mediated mitophagy, and reducing OS [143]. These findings provide support for a gut–mitochondria–brain mechanism by linking microbial modulation with mitophagy and SCFA receptors, but they do not directly demonstrate an epigenetic intermediary within the same model. In addition, while the use of an engineered bacterial strain complicates direct comparison with conventional probiotics, the reported changes in GM composition and SCFA-responsive signaling suggest that the effects may involve multiple interconnected pathways rather than a direct probiotic-mediated activation of mitophagy alone.

In another study conducted by Hafezi et al., a probiotic cocktail containing four native probiotic strains of *Lactobacillus* spp. and *Bifidobacterium* spp. was able to alleviate nickel-induced OS in the brain tissue of nickel-exposed mice by elevating the expression of autophagy genes (ATG13, ATG5, ATG7, and BECN1 genes) and activating autophagy-related pathways [144]. However, because this study used a nickel-induced toxicity model, the findings may reflect the ability of probiotics to counteract toxin-associated oxidative and cellular stress rather than a generalizable effect on autophagy in neurological disorders. Furthermore, increased expression of autophagy-related genes does not by itself establish completion of autophagic flux. Therefore, this study provides evidence for a probiotic–autophagy relationship but does not establish a direct GM–epigenetic–autophagy interplay.

Qi et al. also reported that supplementation with *Lactiplantibacillus plantarum* SG5 alleviated motor deficits and damage to dopaminergic neurons in PD mice by improving mitochondrial quality through the restoration of mitochondrial biogenesis, dynamics, and autophagy-related processes via the GLP-1/PGC-1α pathway [145]. Although the study supports an association between probiotic administration and improved mitochondrial quality control, it does not directly establish an epigenetic mechanism linking the GM to autophagy.

Taken together, these studies suggest that probiotics may influence autophagy and particularly mitophagy through several interconnected mechanisms, including PINK1/Parkin signaling, GLP-1/PGC-1α signaling, SCFA-responsive receptors, OS modulation, and preservation of intestinal barrier integrity. These studies predominantly provide pairwise or indirect evidence, and direct demonstration of a GM–epigenetic–autophagy pathway within the same experimental model remains limited. However, the apparent consistency of the neuroprotective effects should not be interpreted as evidence for a single conserved probiotic–autophagy mechanism. The studies differ substantially in probiotic strain or formulation, host species, disease or injury model, tissue examined, experimental duration, and methods used to assess autophagy. Importantly, most of the available evidence is preclinical, and relatively few studies have directly demonstrated complete autophagic flux in relevant intestinal or neural tissues. Future studies using standardized probiotic preparations, well-characterized GM profiling, sex- and age-balanced experimental designs, dose–response analyses, and direct flux assays will be important for determining whether modulation of autophagy represents a primary mechanism of probiotic-mediated neuroprotection or a secondary consequence of improved metabolic, inflammatory, and mitochondrial homeostasis.

In sum, the evidence supporting probiotic-mediated modulation of autophagy in NPDs and NDs remains predominantly preclinical, with findings from cell-based assays and animal models providing mechanistic plausibility but limited clinical validation. Although some studies provide evidence of effects on mitophagy and mitochondrial quality control, differences in probiotic strains, disease models, and autophagy assessment approaches limit generalizability. In addition, direct evidence linking probiotic-induced epigenetic alterations to autophagy within the same experimental system remains scarce. Thus, the therapeutic potential of probiotics is promising but needs validation in well-designed clinical studies and experiments directly examining autophagic flux and epigenetic mechanisms.

### 6.2. Postbiotics

Postbiotics are functional bioactive compounds produced by beneficial gut microbes in a matrix during fermentation and can contribute to improving health by enhancing blood–brain barrier (BBB) integrity [146,147], regulating inflammation and OS [148,149], and modulating autophagy [150]. Among them, SCFAs can exert protective effects by inducing autophagy in intestinal epithelial cells and stabilizing hypoxia-inducible factor 1-alpha (HIF-1α) [151]. Urolithin A, another GM-derived metabolite generated through microbial metabolism of ellagitannins and ellagic acid present in foods such as nuts, berries, and pomegranate, also supports health and may protect against age-related dysfunction by improving mitophagy, mitochondrial function, and anti-inflammatory actions [152]. However, the effects of postbiotics on autophagy are not uniformly stimulatory and may vary according to the specific metabolite, concentration, exposure duration, cellular metabolic state, and pathological context. Moreover, evidence derived from individual metabolites or purified compounds should not necessarily be equated with the effects of complex postbiotic preparations or intact microbial communities.

Certain GM-derived metabolites like butyrate (an epigenetic regulator), which may be increased following administration of *S. boulardii*, are capable of improving PRKN recruitment to mitochondrial fractions and thereby attenuating mitochondrial mass [153]. Butyrate has also demonstrated the ability to increase the expression of genes relevant to mitochondrial fission and mitophagy, such as dynamin-related protein 1 (DRP1), PINK1, LC3, and PTEN [154]. It has been shown that high glucose-induced ROS and suppressed PRKAA/AMPKα enhance the formation of the RELA/p65-histone deacetylase 8 (HDAC8) complex, and thereby reduce the expression of the PRKN protein by binding to the PRKN promoter region. However, sodium butyrate (SB) is capable of restoring PRKN expression and attenuating high glucose-induced mitophagy dysfunction and neuronal apoptosis by blocking RELA nuclear translocation and suppressing HDAC8 in the nucleus of human-induced pluripotent stem cell-derived neurons, SH-SY5Ys, and streptozotocin (STZ)-induced diabetic mice [155]. These findings illustrate a potential connection between microbial metabolites, epigenetic regulation, mitochondrial quality control, and neuronal survival. Nevertheless, the use of SB or purified butyrate in experimental models does not necessarily reproduce the concentration, bioavailability, or tissue exposure achieved through physiological microbial production. Thus, further in vivo studies are needed to determine the extent to which GM-derived butyrate directly mediates these effects.

Kakoty et al. established PD in Wistar rats using preformed fibrillar form of α-syn and reported that combination therapy with trehalose (4 g/kg, orally), and SB (300 mg/kg, orally) alleviated motor and cognitive deficits by inhibiting pro-inflammatory cytokines, increasing dopamine levels and histone H3 acetylation, and promoting the expression of certain autophagy related genes and proteins [156]. Although this study provided evidence linking epigenetic regulation and autophagy in a PD animal model, the direct role of the GM and the clinical relevance of these findings in humans remain to be established.

Although these findings provide evidence for a potential interaction between HDAC inhibition, histone acetylation, and autophagy in PD, the combined administration of trehalose and SB makes it difficult to attribute the observed effects specifically to butyrate. In addition, increased expression of autophagy-related genes or proteins does not necessarily demonstrate enhanced autophagic flux.

In an in vitro study using rat dopaminergic PC12 (pheochromocytoma 12) cells, Zhang et al. found that SB protected against rotenone-induced toxicity via the activation of autophagy and by increasing the levels of histone 3 lysine 9 acetylation (H3K9Ac) and histone 3 lysine 27 acetylation (H3K27Ac) at the PGC-1α promoter region [157]. This study provided evidence for an epigenetic–autophagy association, but not a direct GM-mediated mechanism. Moreover, the use of a transformed neuronal cell line may not fully recapitulate the cellular complexity of PD in vivo.

In another study, blood and fecal levels of propionic acid, along with the fecal abundance of *Akkermansia muciniphila*, were reduced in patients with AD compared with healthy individuals. Complementary in vivo and in vitro studies reported that propionate, a regulator of histone acetylation, enhanced cognition in mice by reducing the expression of the mitochondrial fission protein (DRP1) through GPR41, enhancing PINK1/PARKIN-mediated mitophagy through GPR43, thereby promoting normal mitochondrial function [158]. While these findings support the proposed mechanism across different experimental systems, the efficacy of propionic acid in improving cognitive impairment in humans remains to be established.

Sun et al. also reported that butyrate-modified hyaluronic acid increased the survival of dopaminergic neurons and attenuated α-synuclein aggregation by enhancing PINK1/Parkin-mediated mitophagy, improving GM composition, and elevating SCFA concentrations, especially butyrate in PD mice [159]. This provides evidence connecting GM alterations and mitophagy, but the role of epigenetic regulation has not been directly examined.

Together, these findings suggest that different SCFAs may influence mitochondrial quality control through partially distinct mechanisms involving G protein-coupled receptor (GPCR) signaling, histone acetylation, and PINK1/Parkin pathways. However, the use of different disease models, delivery systems, and experimental concentrations makes direct comparison between these studies difficult. In particular, the effects observed with butyrate-modified biomaterials may not be directly comparable with those produced by orally or physiologically generated SCFAs.

In addition to butyrate, acetate may exert neuroprotective effects by modulating the autophagy process. For example, acetate, as an epigenetic modulator, could diminish metabolic abnormalities, spatial learning and memory deficits, neuroinflammation, and neuronal death by promoting hippocampal mitophagy through restoration of LC3–translocase of outer mitochondrial membrane 20 (TOMM20) colocalization and increasing the activity of the PINK1/Parkin pathway in a diabetic cognitive impairment (DCI) mouse model established by a 24-week HFD [160]. This supports an epigenetic metabolite–mitophagy relationship without a direct GM contribution.

Moreover, valeric acid has demonstrated the ability to preserve dopaminergic neurons against rotenone-induced hyperactivation of microglia and astrocytes, attenuate OS and rotenone-mediated autophagic vacuole accumulation, and enhance lysosomal degradation in a rotenone induced PD rat model [161]. Propionate, as another epigenetic regulator, has also been identified to be a neuroprotective agent that alleviates neural degeneration by regulating mitochondrial fission and fusion, processes associated with mitophagy/autophagy, in an in vitro BBB model and an AD mouse model [162]. Importantly, these studies suggest that SCFAs and related metabolites can affect multiple stages of mitochondrial quality control; however, their effects should not be interpreted as evidence that all SCFAs uniformly activate autophagy. Changes in LC3 localization, autophagic vacuoles, or autophagy-related proteins may reflect either increased autophagy initiation or impaired downstream degradation, depending on the experimental context. Evidence of enhanced lysosomal degradation or other direct measures of autophagic flux therefore provides stronger support for increased autophagic activity.

Additionally, alpha-ketoglutarate produced by gut bacteria or *Rhizobium* bacteria (an environmental and plant-associated genus of bacteria) has been found to reduce α-synuclein pathology and diminish dopaminergic neuron degeneration in PD mice. These effects were mediated through the promotion of complement component 1q (C1q)-mediated autophagy, the suppression of pro-inflammatory responses, modulation of polyunsaturated fatty acid (PUFA) metabolism, and alterations in the abundance of the *Lachnospiraceae* NK4A136 group involved in GBA regulation, potentially through epigenetic mechanisms [163]. While this in vivo preclinical study in a mouse model of PD shows links between GM metabolites, GM changes, and autophagy, the proposed epigenetic component remains indirect. This finding further illustrates that GM metabolites may affect autophagy through mechanisms extending beyond SCFAs, including immune signaling and metabolic reprogramming. Nevertheless, the contribution of individual bacterial taxa and metabolites remains difficult to separate because GM-directed interventions can simultaneously alter multiple microbial species and metabolic pathways.

Overall, the available evidence indicates that postbiotics may regulate autophagy and particularly mitophagy through interconnected mechanisms involving HDAC inhibition and histone acetylation, GPCR signaling, AMPK-related metabolic pathways, PINK1/Parkin signaling, mitochondrial dynamics, and inflammatory regulation. Most studies provide pairwise or indirect evidence, and direct demonstration of the complete GM–epigenetic–autophagy pathway remains limited. However, the apparent consistency of these effects should be interpreted cautiously because the studies differ substantially in metabolite identity, dose, route of administration, exposure duration, species, age and disease model, tissue or cell type, and methods used to assess autophagy. Furthermore, many studies rely on changes in autophagy-related proteins or mitochondrial markers rather than direct measurements of complete autophagic flux. Future studies should therefore integrate standardized metabolite exposure, longitudinal assessment of autophagic flux, GM profiling, metabolomic measurements, and disease-specific models to determine whether postbiotic-mediated modulation of autophagy represents a primary mechanism of neuroprotection or a secondary consequence of improved mitochondrial, metabolic, and inflammatory homeostasis.

Generally, available evidence supporting postbiotic-mediated modulation of autophagy in NPDs and NDs is mainly preclinical. Studies of SCFAs and other microbial metabolites provide mechanistic evidence linking epigenetic regulation, mitochondrial quality control, and autophagy, but much of this evidence is based on cell-based and animal models using purified metabolites or specialized delivery systems. Additionally, direct evidence that these effects are mediated by GM-derived metabolites under physiological conditions remains limited, particularly with respect to the complete GM–epigenetic–autophagy pathway. Differences in metabolite concentrations and methods used to assess autophagy also limit comparisons across studies. Therefore, although postbiotics represent promising therapeutic candidates, the studies described above do not establish clinical efficacy in humans, and further evidence from human studies is needed to support their potential clinical applications.

### 6.3. Prebiotics

Prebiotics are substrates that are selectively utilized by host microorganisms and confer a health benefit [164]. Unlike direct administration of probiotic microorganisms or isolated GM metabolites, prebiotic interventions primarily act by modifying the intestinal microbial ecosystem and, consequently, the production of GM metabolites. Therefore, their effects on autophagy are likely to be indirect and may depend strongly on the baseline GM, dietary context, host metabolic state, and disease model. In a preclinical study, pretreatment with lactulose and trehalose improved short-term memory and learning retrieval in an AD mouse model by reducing neuroinflammation and enhancing the function of macroautophagy and the CMA pathway [165].

In another preclinical study, intragastric gavage administration of gac oil, camellia oil, and their mixture mitigated cognitive decline in aluminum chloride (AlCl_3_)-induced AD model rats by reducing AD-associated brain pathology, enhancing antioxidant and anti-inflammatory responses, and improving autophagy by increasing the abundance of SCFA-producing bacteria, including the *[Eubacterium]_coprostanoligenes*_group and *Lactobacillus johnsonii* [166]. These findings link GM regulation with autophagy, but there is no direct demonstration of an epigenetic intermediary. Although these studies support a potential relationship between GM modulation and autophagy, the interventions differ substantially in chemical composition and mechanism, making it difficult to determine whether the observed effects resulted directly from prebiotic activity, changes in SCFA production, or secondary improvements in inflammation and OS.

In an in vivo and in vitro study, piperine diminished α-synuclein aggregation in the colon and substantia nigra, mitigated the loss of dopaminergic neurons, and improved autonomic movement and gastrointestinal dysfunctions in 6-hydroxydopamine (6-OHDA)-induced PD rats by promoting the fusion of autophagosomes and lysosomes in neuronal cells and enhancing GBA-associated autophagy through inhibiting the phosphoinositide 3-kinase (PI3K)/protein kinase B (AKT)/mTOR pathway [167]. Because piperine is a plant-derived bioactive compound rather than a conventional prebiotic, this finding is better interpreted as evidence for a dietary bioactive-mediated modulation of the GM–autophagy axis rather than as direct evidence of a prebiotic effect. Other studies reported that piperine modulates epigenetic codes by increasing miR-29c, which suppresses DNA methyltransferase 3B (DNMT3B), and inhibiting histone acetylation [168]. However, these findings come from separate studies and there is no direct focus on a GM–epigenetic–autophagy pathway in the same model.

Palmatine, a berberine alkaloid and an organic heterotetracyclic compound, has also been reported to mitigate Tau hyperphosphorylation, Aβ accumulation in the brain, and the death of hippocampal neurons in AD model rats by enhancing the expression of genes involved in autophagy, including *LC3*, *BECN1*, and *AMPK*, while inhibiting the expression of *mTOR* and sequestosome 1 (*p62*), and elevating the content of SCFAs [169]. Although this animal study links changes in SCFA content with autophagy-related outcomes, the epigenetic component remains indirect, as changes in SCFA content alone do not establish that GM-derived metabolites mediate the observed autophagy-related effects through epigenetic modifications.

These findings suggest that dietary phytochemicals may simultaneously influence GM metabolites, epigenetic pathways, and autophagy; however, the relative contribution of direct host signaling versus GM-mediated effects remains difficult to distinguish.

However, one study reported that chitosan oligosaccharide increased dopaminergic neurons in the substantia nigra (SN) and ameliorated dyskinesia in a PD mouse model by decreasing bacterial metabolites—valeric acid, attenuating inflammation, and activating autophagy [170]. This study demonstrates that a GM metabolite can modulate the autophagy-related effects of a dietary intervention, but it does not establish a direct three-way mechanism.

This finding is particularly important because it demonstrates that GM metabolites do not necessarily exert uniformly beneficial effects on autophagy or neuroprotection. The apparent discrepancy may reflect differences in metabolite concentration, microbial context, disease model, exposure duration, or interactions between individual metabolites and the primary intervention. Thus, changes in SCFA abundance should not automatically be interpreted as beneficial consequences of a GM-targeted intervention.

Other plant-based compounds, such as phytic acid and fucoidan, also exhibited epigenetic effects by decreasing age-related DNA hypomethylation of both long interspersed nuclear element-1 (LINE-1) and short interspersed nuclear elements (SINEs) through increasing the expression of autophagy related genes and sirtuins (SIRT1 and SIRT6) [171,172,173]. Kuwanon V, a plant-based prenylated flavonoid with antioxidant, anti-inflammatory, antimicrobial, and neuroprotective effects, was reported to act through increasing miR-181a expression [174] which is downregulated in ASD [103]. However, these compounds should be distinguished from classical prebiotics because their biological effects may involve direct host-cell signaling in addition to, or independently of, GM modulation.

In an in vivo study, administration of *Phyllanthus emblica* polysaccharides alleviated cognitive impairment in aluminum chloride (AlCl_3_)-induced rats by elevating the abundance of SCFAs and SCFA-producing gut bacteria, particularly the Christensenellaceae_R-7_group, inhibiting pro-inflammatory cytokines (tumor necrosis factor-alpha (TNF-α), interleukin-6 (IL-6), and interleukin-1 beta (IL-1β)) and malondialdehyde (MDA), and enhancing anti-inflammatory cytokines (IL-4 and IL-10) and antioxidant enzymes (superoxide dismutase (SOD), catalase, and glutathione peroxidase (GPx) [175]. Mechanistically, *Phyllanthus emblica* polysaccharides could promote autophagy-related processes by increasing the expression of autophagy-related proteins (ATG5, BECN1, and LC3B) and low-density lipoprotein receptor-related protein 1 (LRP1), while suppressing AD-related proteins, including beta-secretase 1 (BACE1), amyloid precursor protein (APP), Aβ, and Tau phosphorylated at serine 404 [175]. Although this study links GM/metabolite changes with autophagy in rats, the involvement of epigenetic mechanisms has not been directly examined, particularly in a human-relevant context. However, the increase in autophagy-related proteins does not by itself establish increased autophagic flux, and the simultaneous reduction in OS and inflammation makes it difficult to determine whether autophagy activation is a primary mechanism or a downstream consequence of improved cellular homeostasis.

According to another report, *WenDan Decoction*, a classical traditional Chinese medicine formula, could attenuate depressive-like behaviors in high-fat diet (HFD) mice by reducing BBB disruption, inhibiting pro-inflammatory metabolites and the PI3K/AKT/mTOR pathway, and restoring autophagic flux, which was associated with decreased abundance of potentially inflammation-associated taxa,, such as *Faecalibaculum* and *Colidextribacter*, and an enhanced abundance of other taxa, including *Ligilactobacillus* and *Alistipes*, which may contribute to the production of bioactive microbial metabolites [176]. This study provides in vivo preclinical evidence from an HFD mouse model. It indicates stronger evidence for a GM–autophagy relationship because alterations in GM composition were accompanied by functional restoration of autophagic flux; however, an epigenetic intermediary has not been directly demonstrated. In contrast to studies relying primarily on autophagy-related protein expression, the reported restoration of autophagic flux provides stronger evidence for functional regulation of the autophagy pathway. However, the complex composition of this herbal formulation prevents attribution of the effect to a single prebiotic component or microbial taxon.

Furthermore, whey protein hydrolysate could alleviate memory dysfunction in amyloid precursor protein/presenilin 1 (APP/PS1) mice by elevating the abundance of *Dubosiella* and reducing the abundance of *Bacteroides* and *norank_f__Ruminococcaceae*, while enhancing SCFA production, promoting autophagy of neurotoxic Aβ (as indicated by changes in ATG4b, Nsfl1c, Tcf20, Nr2f1, and Trappc9, and reduced expression of Krt1), synaptogenesis, neurotransmitter delivery, and antioxidant defense [177]. This study provides in vivo preclinical evidence from an APP/PS1 mouse model. These findings support a GM–metabolite–autophagy relationship, but there is no direct demonstration of an epigenetic mechanism. Moreover, changes in autophagy-related genes do not, by themselves, establish enhanced autophagic flux.

Again, these findings support an association among dietary intervention, GM remodeling, GM metabolites, and autophagy, but the simultaneous changes in several biological pathways make it difficult to establish a direct causal sequence from GM alteration to autophagy.

Lactoferrin, a multifunctional iron-binding glycoprotein of the transferrin family, could also attenuate brain and intestinal pathological damage in D-galactose (D-gal)-induced aging mice by attenuating OS and inflammatory mediators, regulating the PI3K/Akt/m-TOR pathway to restore autophagy-related activity (elevating LC3II/I and BECN1 expression), restoring the *Firmicutes*/*Bacteroidetes* ratio, and elevating the concentrations of microbial-derived metabolites with potential epigenetic activity [178]. This study provides in vivo preclinical evidence from a D-gal-induced aging mouse model and links GM-related alterations, GM metabolites, and autophagy, but the epigenetic link remains indirect. Lactoferrin can also modulate DNA methylation and mitigate ROS [179] and enhance autophagy through a BECN1-dependent mechanism and B-cell lymphoma 2 (BCL2) inhibition in osteoblasts [180], while it can also protect against methamphetamine toxicity and neurodegeneration by promoting autophagy and preserving mitochondrial mass [181]. These additional studies show separate epigenetic–autophagy associations rather than a direct three-way interplay.

These findings illustrate the complexity of interpreting GM-associated interventions because lactoferrin can exert direct host-cell effects in addition to modifying the intestinal environment. Moreover, the use of the *Firmicutes*/*Bacteroidetes* ratio as an indicator of GM improvement should be interpreted cautiously because this broad taxonomic ratio does not necessarily reflect functional GM changes or metabolite production.

### 6.4. Ketogenic Diet (KD)

The KD, a specific diet containing high fat and very low carbohydrate levels, is another remedy that leads to remarkable alterations in protein and fat metabolism, modulates GM composition and its metabolites, and elevates the production of ketone bodies, which may act as epigenetic regulators, especially via HDAC inhibition [182,183,184,185]. These effects may contribute to neurological and metabolic health and may influence disease-related pathways involving autophagy. Because the KD simultaneously alters nutrient availability, host metabolism, microbial composition, microbial metabolites, and ketone-body production, its effects cannot be attributed exclusively to prebiotic modulation of the GM. This metabolic complexity is an important consideration when interpreting its effects on autophagy. Exogenous ketone bodies are also capable of regulating autophagy both in vitro and in vivo [186]. For example, the ketone body, D-β-hydroxybutyrate, can elevate mitochondrial membrane potential, modulate the nicotinamide adenine dinucleotide (NAD^+^)/reduced nicotinamide adenine dinucleotide (NADH) ratio, increase forkhead box protein O1 (FOXO1), forkhead box protein O3a (FOXO3a), and peroxisome proliferator-activated receptor gamma coactivator 1-alpha (PGC-1α) nuclear levels in an SIRT2-related fashion and activate autophagy, mitophagy, and mitochondrial biogenesis in neuronal cells [187]. These findings provide evidence of a ketone body–autophagy relationship in a neuronal cell model but do not establish direct GM involvement. The contribution of epigenetic regulation should also be distinguished from other SIRT2-related effects. This shows a ketone body–epigenetic/autophagy correlation, but not direct evidence of GM involvement. KD may also promote the formation of autophagosomes and autophagy, enhance concentrations and utilization of ketone bodies (β-hydroxybutyrate and acetoacetate) and their transporting proteins in the hippocampus [188]. Moreover, KD attenuates the detrimental impact of stress on gut mitochondrial function and biogenesis by enhancing baseline autophagy and diminishing the inflammatory response and OS [189]. KD is also capable of exerting neuroprotective effects by reducing the NLRP3-mediated inflammatory response in microglial cells through HDAC3 inhibition and increasing mitophagy-dependent protein acetylation [190]. Collectively, these findings support links between dietary metabolism, epigenetic regulation, and autophagy, while direct evidence demonstrating all three components within the same model remains limited.

Overall, the evidence suggests that prebiotic and broader dietary interventions may influence autophagy through interconnected effects on the GM, GM metabolites, inflammatory signaling, OS, nutrient sensing, and epigenetic regulation. However, the available studies are highly heterogeneous with respect to intervention composition, dose, duration, route of administration, animal species, age, sex, disease model, tissue examined, and GM profiling methods. Importantly, several studies infer autophagy activation from changes in LC3, BECN1, ATG proteins, or related genes, whereas fewer directly demonstrate enhanced autophagic flux or lysosomal degradation. In addition, changes in the abundance of individual bacterial taxa or SCFAs do not necessarily establish a causal relationship with altered autophagy. Therefore, the neuroprotective effects observed with these interventions may reflect the combined effects of GM remodeling, metabolite production, metabolic adaptation, anti-inflammatory activity, and direct host signaling rather than activation of autophagy alone. Future studies using standardized interventions, longitudinal microbiome and metabolome profiling, sex- and age-balanced models, dose–response designs, and direct measurements of autophagic flux will be important for determining the specific contribution of the GM–autophagy pathway to their therapeutic effects.

Collectively, the existing literature supporting prebiotic and dietary interventions for modulating autophagy in NPDs and NDs is predominantly preclinical and varies considerably across interventions. Some studies, particularly those reporting restored autophagic flux, provide stronger functional evidence than studies relying solely on autophagy-related protein expression. However, many interventions discussed in this section are complex dietary compounds or formulations whose effects cannot be attributed specifically to prebiotic activity or GM-mediated mechanisms. Direct evidence linking GM-induced epigenetic changes to autophagy within the same experimental system remains limited, and the evidence presented here is preliminary and does not yet establish clinical efficacy in humans with NPDs or NDs. Thus, further studies should distinguish direct host effects from GM-mediated actions and prioritize functional assessment of autophagic flux and validation in human populations.

### 6.5. Methyl Donors and Methyl Rich Diets

GM plays a key role in the host’s one-carbon metabolism by contributing to the biosynthesis or modification of nutrients that serve as methyl donors or support methyl-group availability [191]. These nutrients contribute to regulating DNA methylation, histone methylation, and other epigenetic processes [192,193]. The major GM-derived or GM-modulated methyl donors and related one-carbon nutrients include folate (B9), methionine, choline, betaine, vitamin B12, vitamin B6, riboflavin (B2), and S-adenosylmethionine (SAM) (the universal methyl donor whose synthesis and availability can be influenced by host and microbial metabolism). Methyl donors and methyl-rich diets can exert beneficial effects on health, and their deficiency or imbalance has been associated with autophagy dysfunction and disease pathogenesis, through various mechanisms, including epigenetic aberrations [194,195,196]. However, the relationship between methyl-donor availability, epigenetic regulation, and autophagy is complex and is not necessarily linear. The effects may depend on the specific nutrient, dose, duration of supplementation, baseline nutritional status, tissue, disease model, and underlying metabolic state. For example, Tripathi et al. found that hyperhomocysteinemia (HHcy) impaired autophagic flux by disrupting lysosomal function and inducing endoplasmic reticulum (ER) stress, hence elevating cellular vulnerability to oxidative injury [197]. However, vitamin B co-treatment protected against HHcy-induced cellular damage by restoring autophagic flux, reducing ER stress, and reversing lysosomal impairment [197]. This study provides relatively strong evidence for restoration of functional autophagy because autophagic flux and lysosomal function were directly evaluated. However, the involvement of GM or a direct epigenetic mechanism was not examined in this model. It also illustrates that the beneficial effect of B vitamins may result from correction of a metabolic disturbance rather than from a universal stimulatory effect on autophagy. Cui et al. engineered a tetrahedral DNA nanostructure carrying vitamin B12 (a cofactor for methionine synthase) and investigated its ability to mitigate pathological damage in a mouse model of MPTP-induced PD [198]. Their results indicated that vitamin B12-loaded tetrahedral framework nucleic acid improved PD motor symptoms by restoring autophagy more effectively than free vitamin B12 both in vivo and in vitro by promoting the clearance of abnormal protein accumulations [198]. This constitutes preclinical evidence from animal and cellular models, rather than evidence from human studies. This study shows a vitamin B12–autophagy association, but not a direct GM–epigenetic–autophagy interaction. Although these findings support a potential role of vitamin B12 in restoring autophagy and protein clearance, the use of a specialized nanostructured delivery system limits direct comparison with conventional dietary B12 supplementation. Thus, the observed effects may reflect both vitamin B12 activity and enhanced cellular delivery.

In contrast, Ren et al. reported that folic acid (a folate-derived methyl-group donor) supplementation may attenuate autophagy-related apoptotic cell death in rat brain neural cells and HT-22 neurons through the p53/mTOR signaling pathway [199]. This research represents preclinical evidence involving rat brain neural cells and an in vitro neuronal cell model. This study primarily shows a folate–autophagy correlation, without directly establishing GM involvement. This finding, together with the effects of B-vitamin treatment in HHcy, indicates that different components of one-carbon metabolism can exert distinct effects on autophagy depending on the cellular and pathological context. Importantly, modulation of autophagy-related apoptosis should not necessarily be interpreted as direct enhancement of autophagic flux.

In another study conducted by Agam et al., choline (an important source of methyl groups) supplementation for two weeks alleviated repetitive and anxiety-like behaviors, improved social behavior in male offspring of methylenetetrahydrofolate reductase (MTHFR)-deficient mothers, and normalized alterations in cortical autophagy markers (p62 and the LC3-II/LC3-I ratio), suggesting that autophagy modulation contributes to its observed effects on autism-like phenotypes [200]. Although the findings of this study link one-carbon metabolism with autophagy-related changes, neither GM alterations nor epigenetic changes were directly measured as mediators.

Because this model involved maternal MTHFR deficiency and male offspring, the findings may reflect interactions between developmental one-carbon metabolism, sex, genetic background, and autophagy. Moreover, normalization of LC3-II/LC3-I and p62 levels provides evidence of altered autophagy-related signaling but does not by itself establish whether autophagic flux was increased or restored.

Meng et al. reported that betaine (a direct methyl-group donor generated by choline oxidation) also acts as an autophagy inducer in microglial HMC3 cells via the PI3K/AKT pathway to attenuate the toxicity of amyloid-beta in human SH-SY5Y cells [201]. This study represents in vitro cellular evidence involving HMC3 microglial cells and SH-SY5Y neuronal cells. This provides evidence for a methyl donor–autophagy association without a direct focus on GM or epigenetic mediation. However, this finding was obtained using an in vitro cellular model and therefore cannot be directly extrapolated to the intact brain or to the effects of dietary betaine supplementation in vivo. In addition, the concentration and exposure duration used experimentally may differ substantially from physiological exposure following dietary intake.

Notably, these vitamins, as well as choline and betaine, are involved in one-carbon metabolism and can influence DNA methylation across multiple tissues, including the brain. The GM can synthesize certain B vitamins, including folate and, in some microbial species, vitamin B12, while also extensively metabolizing choline and other methyl-donor-related nutrients, thereby influencing host one-carbon metabolism and epigenetic regulation [202]. Nevertheless, the contribution of the GM to host methyl-donor availability is highly dependent on microbial composition, dietary substrate availability, intestinal absorption, and host metabolic capacity. Therefore, changes in circulating or tissue concentrations of methyl donors cannot necessarily be attributed to GM activity alone. Although the GM–one-carbon–epigenetic connection is biologically plausible, direct evidence linking these microbial effects to autophagy within the same experimental model remains limited.

Collectively, the available evidence suggests that methyl-donor availability may influence autophagy through interconnected metabolic, epigenetic, lysosomal, and nutrient-sensing pathways. However, the direction of this relationship is context-dependent: B-vitamin supplementation can restore impaired autophagic flux under hyperhomocysteinemic conditions, whereas folate, choline, and betaine have been associated with different patterns of autophagy-related signaling in specific experimental models. Differences in species, genetic background, age, sex, disease model, nutritional status, tissue or cell type, dose, duration of supplementation, and methods used to assess autophagy may contribute to these divergent findings. Furthermore, because one-carbon metabolism simultaneously affects DNA methylation, histone methylation, redox homeostasis, and amino-acid metabolism, changes in autophagy may represent one component of a broader metabolic response rather than an isolated mechanism. Future studies should therefore integrate GM composition, one-carbon metabolite concentrations, epigenetic measurements, and direct assays of autophagic flux to determine how GM-associated methyl-donor metabolism contributes to autophagy regulation in NPDs and NDs.

In sum, evidence linking methyl-donor interventions to autophagy regulation in NPDs and NDs is predominantly preclinical and mechanistically heterogeneous. Although some studies directly assess autophagic flux, most do not simultaneously examine GM alterations, epigenetic changes, and autophagy within the same experimental system. Furthermore, the effects of methyl donors rely on nutritional status, disease context, and metabolic conditions, limiting the generalizability of findings across interventions and disorders. Although their therapeutic potential is promising, insufficient validation highlights the need for integrated studies combining microbiome profiling, one-carbon metabolite measurements, epigenetic analyses, and functional autophagy assays, alongside clinical validation.

### 6.6. Fecal Microbiota Transplant (FMT)

FMT or stool transplant is a process of transferring fecal bacteria and other microbes from a donor to a recipient to modulate the recipient’s GM and potentially improve disease-associated phenotypes; it can also be used experimentally to investigate the contribution of the GM to disease mechanisms [203,204]. FMT has been found to induce intestinal mucosal protective autophagy and mitigate gut barrier damage in infected piglets by altering GM composition, including increasing the abundances of potentially beneficial bacteria such as *Lactobacillus* and *Succinivibrio*, which may contribute to the production of microbial metabolites with epigenetic activity, together with reductions in *Enterobacteriaceae* and *Proteobacteria* [205]. Although this study indicates a connection between GM and autophagy, the epigenetic role was not directly demonstrated. However, the association between these microbial changes and autophagy does not by itself establish that specific taxa or microbial metabolites directly mediate the observed autophagic response. Moreover, findings from infected piglets may not be directly extrapolated to other species or to chronic neurological disorders because host species, disease context, donor GM, recipient GM, and FMT protocol can substantially influence the outcome. This represents in vivo animal evidence, rather than evidence from human studies, and the proposed contribution of epigenetic mechanisms remains hypothetical.

Liu et al. reported that FMT could also exert neuroprotective effects against manganese (Mn)-induced neurotoxicity in mouse brain by suppressing the expression of key proteins, including BECN1, LC3B, and PINK1, associated with autophagy and mitophagy in the hippocampus, while regulating the apelin pathway [206]. This study primarily shows a GM–autophagy relationship, without direct evidence of epigenetic involvement.

Importantly, the reduction in the levels of these autophagy-related proteins should not necessarily be interpreted as inhibition of autophagic flux, because changes in BECN1, LC3B, or PINK1 expression alone cannot distinguish reduced autophagy initiation from enhanced completion and degradation of autophagic cargo. Nevertheless, the neuroprotective effects reported in this FMT study highlight that therapeutic benefits do not necessarily require increased expression of autophagy-related proteins. Depending on the pathological context, reducing excessive or dysregulated autophagy-related responses may be beneficial, although the observed changes do not establish that autophagy itself was directly inhibited.

Thus, the apparently divergent effects of FMT on autophagy in the intestinal and brain models may reflect differences in tissue, disease model, host species, microbial composition, and the stage or type of autophagy being assessed rather than a uniform effect of FMT on the autophagic pathway. These findings show the need to avoid treating autophagy enhancement as a universal therapeutic objective; instead, interventions should aim to restore appropriate autophagic function while accounting for disease-specific requirements and the balance between autophagosome formation and lysosomal degradation.

In addition, FMT effects are highly dependent on donor characteristics, microbial engraftment, recipient baseline GM, administration route, frequency, and treatment duration, which may contribute to variability among studies. Direct assessment of autophagic flux, together with longitudinal microbiome and metabolome profiling, would therefore be important for determining whether GM restoration is causally linked to autophagy-related outcomes. Future studies should also assess DNA methylation, histone modifications, or non-coding RNAs when proposing epigenetic mechanisms, rather than inferring epigenetic involvement solely from changes in GM composition or microbial metabolites.

Overall, evidence supporting FMT-mediated modulation of autophagy in neurological disorders remains limited and predominantly preclinical. Although studies suggest that FMT can influence autophagy-related processes in both intestinal and brain tissues, the divergent findings and limited use of direct autophagic flux measurements restrict mechanistic interpretation. Furthermore, evidence directly linking FMT-induced microbial changes to epigenetic regulation and autophagy within the same experimental system is lacking in the studies discussed. Therefore, FMT remains a promising experimental approach, but its therapeutic relevance to NPDs and NDs requires further validation in disease-specific models and well-designed clinical studies.

## 7. Conclusions and Future Perspectives

In summary, the autophagic–lysosomal pathway contributes to cellular homeostasis and, in many contexts, neuroprotection by clearing protein aggregates and maintaining cellular homeostasis, thereby potentially counteracting the pathogenesis of NPDs and NDs. Furthermore, its role in sustaining homeostasis in peripheral organs, including the gut, can influence both the onset and progression of these diseases. Understanding the molecular mechanisms underlying the association between impaired autophagy and gut dysbiosis and their interactions with other etiological factors, such as epigenetic alterations involved in disease progression, is critical for developing new therapeutic strategies. It should be noted that autophagy is not uniformly protective, and its beneficial or detrimental effects may depend on the disease context, cell type, stage of disease, and balance between autophagosome formation and lysosomal degradation. Therefore, therapeutic strategies should aim to restore balanced and functional autophagic flux rather than simply enhance or inhibit autophagy. Although most of the GM-mediated therapeutic interventions discussed in this review target or influence histone modifications, it is important to note that histone modifications and DNA methylation are functionally interconnected epigenetic mechanisms that cooperatively regulate chromatin structure and gene expression. Histone modifications, particularly histone H3 lysine 9 methylation (H3K9me2/3), can recruit DNA methyltransferases (DNMTs) to specific genomic regions, thereby promoting de novo DNA methylation, whereas activating histone marks such as H3K4me3 inhibit DNMT recruitment and protect promoters from DNA methylation. Consequently, alterations in histone modification patterns can directly influence the establishment and maintenance of DNA methylation landscapes [207,208,209]. Regarding miRNAs, although several lines of evidence indicate that autophagy-related miRNAs are affected by GM [105,106] and are involved in the pathogenesis of NPDs and NDs (Table 2), experimental evidence directly linking GM modulation or GM-derived metabolites to the normalization of these miRNAs remains limited compared with that for other epigenetic mechanisms. Therefore, the role of GM–miRNA interactions in the epigenetic regulation of autophagy represents an emerging area of research that warrants further investigation.

Despite growing evidence supporting the interactions among autophagy, the GM, and epigenetic regulation in NPDs, several challenges remain to be addressed. One major limitation is the complexity of the interplays within and between the elements of this axis, which hinders the determination of causal relationships and the underlying molecular mechanisms. In addition, findings from animal models are difficult to translate directly to humans due to differences in physiology, genetics, environmental factors, and GM composition. The heterogeneity of NPDs, variations in study design, sample collection, analytical methods, and patient populations make the interpretation and comparison of results across studies difficult. Moreover, the lack of standardized biomarkers, together with the diversity of biomarkers used across studies to assess autophagic activity, gut microbial dysbiosis, and epigenetic alterations restricts the design and developing reliable diagnostic and therapeutic strategies. In particular, focusing on static autophagy-related markers without direct evaluation of lysosomal degradation or autophagic flux may result in misinterpretation of autophagy activity. It should be noted that direct experimental evidence simultaneously linking the GM, epigenetic regulation, and autophagy within the same experimental system remains scarce. Most available studies investigate pairwise interactions between these components, whereas the proposed three-way interplay is often inferred from complementary findings across different experimental models or mechanistic pathways. This major challenge limits the ability to establish causal relationships and determine how these components interact to contribute to NPDs and NDs. Future studies should investigate these three components concurrently within integrated experimental designs to validate the proposed mechanisms and clarify their disease-specific relevance.

Moreover, future research should focus on integrating multi-omics approaches, including genomics, epigenomics, transcriptomics, metabolomics, and microbiomics, to provide a comprehensive understanding of the autophagy–GM–epigenome axis in NPDs. Nevertheless, longitudinal, large-scale clinical studies are needed to clarify temporal relationships and identify disease-specific molecular signatures. Recent advances in single-cell technologies, artificial intelligence, and systems biology may help uncover novel regulatory networks and therapeutic targets. Consequently, future investigations will be better equipped to evaluate the efficacy and safety of GM-based interventions, such as probiotics, prebiotics, dietary modifications, and FMT, in restoring autophagic function and epigenetic homeostasis with greater precision. A more comprehensive understanding of these mechanisms may also facilitate the development of personalized therapeutic strategies for the prevention and treatment of NPDs.

## Figures and Tables

**Table 2:** Altered miRNAs and affected genes involved in autophagy in neurodegenerative and neuropsychiatric diseases.

Type of miRNA/Disease	Type of Study/Sample	Affected Genes/Main Methods	Main Findings	Ref.
miR-21, miR-26b, miR-106a, miR-224, miR-301b, miR-320a, miR-373, and miR-379/Parkinson’s disease (PD)	Human SH-SY5Y neuroblastoma cell line/Substantia nigra pars compacta and amygdala samples from PD patients vs. controls	Chaperone-mediated autophagy (CMA) proteins LAMP-2A and hsc70/Western blotting (WB) and RT-PCR	Elevations in specific miRNAs reduce LAMP-2A and hsc70 protein levels, thereby intracellular α-synuclein accumulation due to impairment of CMA	[107]
miR-320a/PD	SH-SY5Y cells with human origin	Hsc70/WB and RT-PCR	Increased expression of miR-320a reduces Hsc 70 expression, which in turn suppresses CMA	[108]
miR-185/PD	MPTP-treated SH-SY5Y cells	BECN1, LC3I/II, p-AMPK and p-mTOR/WB and RT-PCR	Elevated expression of miR-185 diminishes autophagy and apoptotic cell death of dopaminergic neurons	[109]
miR-124/PD	MPTP-treated SH-SY5Y cells and MPTP-treated mice	LC3, p62 and p38/WB	miR-124 suppress expression of p62/p38, promotes autophagy and suppress microglia activation in substantia nigra	[110]
miR-125b-5p/PD	MPP+-induced SH-SY5Y cell model and mice/brain tissue	BDNF-AS, LCI and LCII/RT-PCR. WB and transmission electron microscopy (TEM)	Downregulation of miR-125b-5p and increased expression of BDNF-AS in MPTP-induced PD models is linked to elevation of autophagy and apoptosis	[111]
miR-124/Alzheimer’s disease (AD)	APP/PS1 transgenic mice/brain	BACE1 and ATG5/WB and RT-PCR	miR-124 exerts neuroprotective effect via suppressing BACE 1 and subsequent autophagy regulation	[112]
miRNA-101a/AD	AD patients and APPswe/PS1ΔE9 transgenic mice/plasma	MAPK1, BECN1, and LC3/WB and TEM	Reduced plasma miRNA-101a levels and impaired autophagic vacuoles formation through the MAPK pathway	[113]
miR-17/AD	AD patients and (5xFAD) mice/temporal lobe brain tissue (Brodmann area 38) from AD patients vs. controls	LC3 and autophagy cargo receptor NBR1 critical in Aβ degradation/WB and RT-PCR	Negative correlation between NBR1 expression and miR-17 in human AD microglia/suppressing miR-17 in 5xFAD mouse microglia enhanced Aβ degradation by improving autophagy	[114]
miR-331-3p and miR-9-5p/AD	SY5Y cell line/APPswe/PS1dE9 mice/brain tissues	BECN1, Sqstm1, and LC3b/RT-PCR and WB	Increased expression of miR-331-3p and miR-9-5p in SH-SY5Y cell line disrupted autophagic function and aggravated amyloid plaques generation	[115]
miR-4763-3p/AD	AD-mild cognitive impairment (MCI) in humans and mice/human serum and brain and mouse hippocampus	SQSTM1, LC3B II/I, PI3K, AKT, mTOR, and Bcl2/Microarray, RT-PCR and WB	Age-associated hyperexpression of miR-4763-3p in AD hippocampal colocalizing with Aβ and Tau/miR-4763-3p affects autophagy and cognitive function in AD-MCI mice	[116]
miR-375-3p/AD	SH-SY5Y cells/APP/PS1 TG mice	PS1, ATG14, BECN1, LC3B, MFN2, Grp75, Presenilin 1, and mTOR/WB and RT-PCR	miR375-3p elevated autophagy in AD mice by decreasing presenilin 1 expression and suppressing mTOR phosphorylation	[117]
miR-181a-5p/Autism-spectrum disorder (ASD)	Children with idiopathic ASD and healthy controls/white blood cells	LAMP1, LAMP2, CerS1, SMPD5, COX2, and YKL40/RT-PCR	Elevated mRNA levels of CerS1, SMPD5, COX2, YKL40, LAMP1, and LAMP2 and downregulation of miRNA-181a-5p in ASD subjects vs. controls	[103]
miR-134-5p/depression	Chronic unpredictable mild stress (CUMS) mice/hippocampus	LC3B and MAP-2/Immunofluorescence and RT-PCR	Knockdown of miR-134-5p enhanced AMPK-mediated mitophagy in dendrites, reduced mitochondrial dysfunction, and promoted autophagosome generation	[118]
miR-103-3p/depression	CUMS rats/hippocampus	Wnt/β-catenin, BDNF, CREB, BECN-1, Rab10, and p62/RT-PCR and WB	miRNA-103-3p stimulated Wnt/β-catenin signaling by targeting rab10 to enhance neural plasticity and autophagy	[119]
miR-302b-3p/depression	CUMS mice/prefrontal cortex	ULK1, p62, ATG5, BECN1, and LC3-II/WB and immunofluorescence	Activation of autophagy through miR-302b-3p/ULK1 axis by a therapeutic agent improved depression symptoms	[120]
miR-19b-3p/bipolar depression	Seven unipolar and seven bipolar depressed patients vs. six controls/plasma	mTOR, autophagy-related genes, FoxO, prolactin, p53, and PI3-K/Akt/miRNA Array and qRT-PCR	Hyperexpression of miR-19b-3p and more severe childhood trauma in subjects with bipolar depression vs. unipolar depression	[121]

## Data Availability

No new data were created or analyzed in this study. Data sharing is not applicable to this article.
